# World without borders—genetic population structure of a highly migratory marine predator, the blue shark (*Prionace glauca*)

**DOI:** 10.1002/ece3.2987

**Published:** 2017-05-24

**Authors:** Ana Veríssimo, Íris Sampaio, Jan R. McDowell, Paulo Alexandrino, Gonzalo Mucientes, Nuno Queiroz, Charlene da Silva, Catherine S. Jones, Leslie R. Noble

**Affiliations:** ^1^ CIBIO – U.P. – Research Center for Biodiversity and Genetic ResourcesVairãoPortugal; ^2^ Virginia Institute of Marine ScienceCollege of William and MaryGloucester PointVAUSA; ^3^ Centro Tecnológico del MarFundación CETMARVigoSpain; ^4^ Department of Agriculture, Forestry and FisheriesBranch FisheriesRogge BaySouth Africa; ^5^ Institute of Biological and Environmental SciencesSchool of Biological SciencesUniversity of AberdeenAberdeenUK

**Keywords:** gene flow, highly migratory sharks, nursery areas, panmixia

## Abstract

Highly migratory, cosmopolitan oceanic sharks often exhibit complex movement patterns influenced by ontogeny, reproduction, and feeding. These elusive species are particularly challenging to population genetic studies, as representative samples suitable for inferring genetic structure are difficult to obtain. Our study provides insights into the genetic population structure one of the most abundant and wide‐ranging oceanic shark species, the blue shark *Prionace glauca,* by sampling the least mobile component of the populations, i.e., young‐of‐year and small juveniles (<2 year; *N* = 348 individuals), at three reported nursery areas, namely, western Iberia, Azores, and South Africa. Samples were collected in two different time periods (2002–2008 and 2012–2015) and were screened at 12 nuclear microsatellites and at a 899‐bp fragment of the mitochondrial control region. Our results show temporally stable genetic homogeneity among the three Atlantic nurseries at both nuclear and mitochondrial markers, suggesting basin‐wide panmixia. In addition, comparison of mtDNA CR sequences from Atlantic and Indo‐Pacific locations also indicated genetic homogeneity and unrestricted female‐mediated gene flow between ocean basins. These results are discussed in light of the species' life history and ecology, but suggest that blue shark populations may be connected by gene flow at the global scale. The implications of the present findings to the management of this important fisheries resource are also discussed.

## 1 Introduction

Understanding the processes governing the distribution of marine fisheries resources in time and space is essential for efficient management aimed at long‐term sustainability of populations. Studies on the population structure of highly mobile pelagic marine fishes are often limited by the difficulty of obtaining representative samples across the range of often‐widespread species, and by the expected low level of genetic differentiation among populations due to the potential for high gene flow between distant locations. Highly mobile elasmobranchs are no exception, and the above limitations in studying these charismatic but elusive species are of particular concern given their high vulnerability to population depletion when faced with even low rates of fishing mortality (Musick, Burgess, Cailliet, Camhi, & Fordham, 2000).

One such species is the blue shark *Prionace glauca* (Linnaeus 1758), which is likely the most exploited shark species globally (Camhi, Lauck, Pikitch, & Babcock, 2008). This large (>300 m TL) oceanic pelagic shark is found in temperate and tropical waters in all the world's oceans (Nakano & Stevens, 2008). The species has a wide thermal tolerance, particularly in the adult stage (Vandeperre, Aires‐da‐Silva, Lennert‐Cody, Serrão Santos, & Afonso, 2016; Vandeperre, Aires‐da‐Silva, Santos et al., 2014), and has been caught in waters between 8° and 30° Celsius (Kohler, Turner, Hoey, Natanson, & Briggs, 2002; and references therein). As many other oceanic pelagic sharks, *P. glauca* is highly migratory and can cover distances ~1,000–10,000s km including east–west and north–south trans‐oceanic movements and cross multiple national borders during its life cycle (Kohler et al., 2002; Queiroz, Humphries, Noble, Santos, & Sims, 2012; Sippel et al., 2011; Vandeperre, Aires‐da‐Silva, Lennert‐Cody et al., 2014). Indeed, blue sharks are spatially segregated according to size, sex, and reproductive stage, exhibiting complex movement behaviors associated with feeding, ontogeny, and reproduction (reviewed in Nakano & Stevens, 2008). Nursery areas are generally located at the northern and southern temperate latitudes ~35–45°, where juveniles of both sexes co‐occur (Nakano & Stevens, 2008). As individuals grow and approach sexual maturity, males and females move out of the nursery areas and undertake progressively larger movements (Vandeperre, Aires‐da‐Silva, Lennert‐Cody et al., 2014), eventually spanning the whole ocean basin and exploiting highly productive areas at oceanic frontal zones (Queiroz et al., 2012, 2016).

Blue sharks are extremely abundant in the open ocean and are the most frequent by‐catch of swordfish and tuna longline pelagic fisheries, as well as of recreational fisheries worldwide (Aires‐da‐Silva, Ferreira, & Pereira, 2008; Castro & Mejuto, 1995; Kohler, Casey, & Turner, 1998; Nakano & Seki, 2003). They are thus an important fisheries resource, particularly in Atlantic waters where the fishing effort of high‐seas longline fisheries and reported catches of *P. glauca* are high (Camhi et al., 2008; Queiroz et al., 2012, 2016). However, the current status of the species' stocks remain unclear due to the low quality and limited amount of available catch and effort data (ICCAT 2015; Rice, Harley, & Kai, 2014), a scenario also observed in other oceanic pelagic sharks (Campana, 2016). Not surprisingly, recent stock assessment efforts yielded ambiguous scenarios for Atlantic and Pacific blue shark stocks (ICCAT 2015; Rice et al., 2014), raising issues on the reliability of assessments. Despite being a relatively productive species among elasmobranchs (Cortés et al., 2010), warning signs of regional decreases in Atlantic blue shark catches have already been reported (Aires‐da‐Silva et al., 2008; Hueter & Simpfendorfer, 2008). In addition to the lack of management measures or biological reference points for *P. glauca*, the stock structure of Atlantic blue sharks remains uncertain.

Previous attempts to elucidate the genetic population structure of blue sharks found no significant structure among collections from different North Atlantic locations (Queiroz, 2010) as well as across the North Pacific (King et al., 2015), supporting a single management unit for each region. However, another study found genetic homogeneity across the whole Pacific Ocean at the mitochondrial cytochrome *b* gene (Taguchi, King, Wetklo, Withler, & Yokawa, 2015), questioning the current separation of north versus south stocks within a given ocean basin (ICCAT 2005, 2015; Sippel et al., 2011). All studies so far were based on samples collected opportunistically, a strategy adopted in the majority of the studies dealing with oceanic pelagic sharks given the inherent challenges in sampling these elusive species. However, opportunistic samples may include individuals of mixed origin if adults and subadults originating from distinct genetic stocks co‐occur at a given feeding area, thereby potentially masking all or part of the genetic signal.

Heist (2008) highlights the importance of sampling small juveniles at nursery areas to infer the stock structure of highly mobile pelagic sharks. Nursery areas hold the least‐migratory component of the populations (i.e., neonates and small juvenile sharks) and, consequently, allow some degree of isolation among population units (Heist, 2008). In the case of the blue shark, several nursery areas have been proposed for the North and South Atlantic: off the Azores (Aires‐da‐Silva et al., 2008; Vandeperre, Aires‐da‐Silva, Santos et al., 2014; Vandeperre, Aires‐da‐Silva, Lennert‐Cody et al., 2014), off the western Iberian Peninsula (Stevens, 1990), in the Gulf of Guinea (Castro & Mejuto, 1995), and off western South Africa (Jolly, da Silva, & Attwood, 2013; Petersen, Honig, Ryan, Underhill, & Compagno, 2009; da Silva, Kerwath, Wilke, Meÿer, & Lamberth, 2010). Recently, long‐term tracking of blue sharks tagged around the Azores showed strong site fidelity to this region at all life stages, except adult females for which data were limited, and suggested limited or no mixing of juvenile blue sharks among nursery areas during the first years of life (Vandeperre, Aires‐da‐Silva, Lennert‐Cody et al., 2014).

Given the limited data on the population structure of Atlantic blue sharks, the aim of this study was to (1) test whether there is genetic differentiation among blue shark nurseries in the Atlantic Ocean off western Iberian, Azores, and South Africa; and (2) test the temporal stability of the observed patterns of genetic diversity. To this purpose, 12 nuclear microsatellite loci and a 899‐bp fragment of the mitochondrial DNA control region were used to estimate the levels of genetic diversity and differentiation among sample collections of young‐of‐the‐year (YOY) and small juvenile blue sharks (≤2 year old) within each nursery area, that is, during a stage of limited movement (Kohler & Turner, 2008; Vandeperre, Aires‐da‐Silva, Lennert‐Cody et al., 2014). Thus, we aimed to maximize the power to detect genetic differentiation in the blue shark by avoiding the confounding genetic signal of highly migratory adult sharks of unknown natal origin. Moreover, we compared the genetic diversity of Atlantic blue sharks with that of Indo‐Pacific counterparts making use of previously collected molecular data (Ovenden, Kashiwagi, Broderick, Giles, & Salini, 2009), to infer genetic connectivity among ocean basins in this highly migratory species.

## 2 Methods

### 2.1 Sample collection, DNA extraction, and analysis

**Figure 1:**
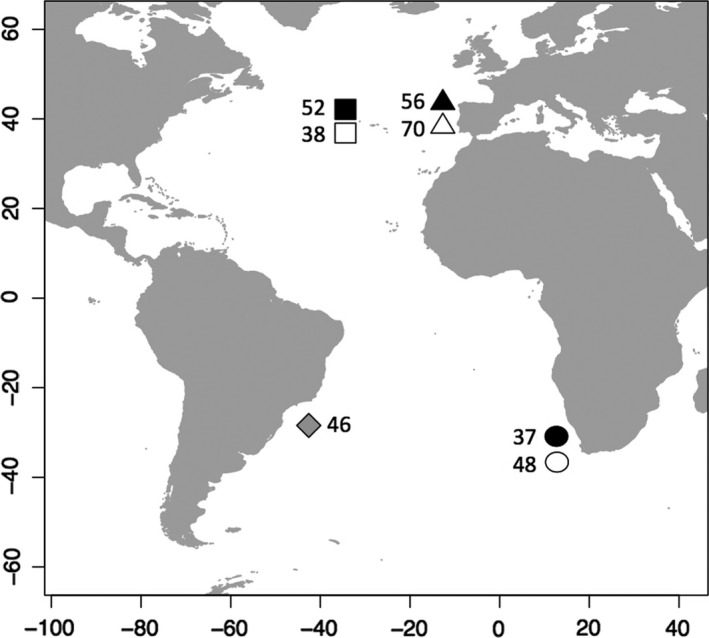
Sampling locations of blue sharks in Atlantic waters. Triangles—Iberian Peninsula, squares—Azores, circles—western South Africa, diamond—western Brazil. Black symbols—individuals from cohorts between 2003 and 2008 (2000s), white symbols—in dividuals from cohorts between 2012 and 2015 (2010s)

The sampling locations correspond to three reported nursery areas of *P. glauca* in the Atlantic Ocean, namely off the Azores (AZ), off western Iberia (IB) and off western South Africa (SA), as well as from Brazil (Figure 1). A total of 302 individual blue sharks smaller than 150 cm fork length (FL) were sampled from the three nursery areas on board commercial fishing vessels operating in the North Atlantic and during pelagic longline research surveys off western South Africa. Research permits for the South African samples were issued by the Department of Agriculture, Forestry, and Fisheries (Republic of South Africa). The sampled individuals corresponded to young‐of‐the‐year (YOY), 1‐ and 2‐year‐old blue sharks (Table 1) according to the age‐at‐length estimates of Skomal and Natanson (2002). Samples at each location were collected at two time intervals corresponding to two groups of cohorts: (1) between 2003 and 2008, hereon designated as 2000s cohorts and (2) between 2012 and 2015, hereon designated as 2010s cohorts (Table 1; Figure S1). Additional samples (*N* = 46) were obtained from observers on board commercial fishing vessels operating in Brazilian waters prior to 2012, although no other biological information (e.g., size or sex) is available. They are thus not considered as samples from juvenile blue sharks, but given the lack of information from specimens from that region, they were analyzed and compared to the remaining sample collections. Tissue samples were obtained from dorsal fins or muscle tissue (~1 cm^3^) preserved in 96% ethanol and stored at room temperature. Total genomic DNA (gDNA) was extracted from each tissue sample using the EasySpin^®^ Genomic DNA Tissue Kit (Citomed, Lisbon), according to the manufacturers' instructions.

**Table 1:** Sample details for Atlantic collections of *P. glauca,* and molecular diversity indices at 12 nuclear microsatellite loci and a 899‐bp fragment of the mtDNA CR

Nursery	Time period	FL range (cm)	Cohorts	mtDNA CR					Nuclear microsatellite loci				
				*N*	*H*	*h*	Pπ	*k*	*N*	*N*_A_	*A*_R_	*H*_*O*_	*F*_IS_
Overall 2,000s		65–126	2,003–2,008	141	32	0.90	.0033	3.00	146	9.8	9.71	0.63	0.03
IB	2,000s	65–120	2,003–2,008	56	21	0.88	.0038	3.45	57	8.5	7.72	0.64	0.04
AZ	2,000s	88–120	2,004–2,006	51	16	0.87	.0040	3.57	52	8.3	7.67	0.62	−0.01
SA	2,000s	85–126	2,006–2,007	34	16	0.93	.0036	3.26	37	7.6	7.42	0.65	0.05
Overall 2,010s		69–140	2,012–2,015	91	27	0.89	.0034	3.05	156	9.7	9.58	0.62	0.06
IB	2,010s	69–140	2,012–2,014	28	13	0.88	.0038	3.43	70	8.6	7.64	0.61	0.07
AZ	2,010s	105–140	2,012–2,014	27	12	0.89	.0039	3.53	38	7.3	7.15	0.62	0.05
SA	2,010s	60–91	2,014–2,015	36	17	0.91	.0039	3.52	48	8.6	7.96	0.62	0.04
Brazil	n.d.	n.d.	n.d.	41	22	0.95	.0035	3.46	46	a	a	a	a

FL, fork length; *N*, sample size; H, no. of haplotypes; *h*, haplotype diversity; Pi, nucleotide diversity; *N*_A_, no. of alleles; *A*_R_, allelic richness; *H*_*O*_, observed heterozygosity; H_*E*_, expected heterozygosity; *F*_IS_, inbreeding coefficient; n.d., no data.

F_IS_ was not significant after FDR correction.

a Diversity indices for the Brazilian collections referring to seven microsatellite loci are presented in Table S3.

The mitochondrial DNA control region (mtDNA CR) was amplified using primers developed for the white shark *Carcharodon carcharias* by Pardini et al. (2001), that is, GWS_F6 5′‐TTGGCTCCCAAAGCCAAGATT‐3′ and PheCacaH2 5′‐CTACTTAGCATCTTCAGTGCC‐ 3′. PCR amplification conditions were optimized for the blue shark and performed in 10 μl reactions including 5 μl of MyTaq HS Mix 2× (Bioline), 3.2 μl of ultra‐pure water, 0.4 μmol/l of each primer, and 1 μl of gDNA. The PCR temperature program included a hot start at 95°C for 5 min followed by 40 cycles of denaturation at 95°C for 1 min, annealing at 65°C for 45 sec, and extension at 72°C for 1 min, and a final extension step at 72°C for 10 min. All amplicons were purified with ExoSap (USB Corporation) following the manufacturer′s guidelines, prior to shipping and sequencing at Macrogen Europe (Macrogen Inc., The Netherlands). Both the forward and reverse strands were sequenced, and quality and accuracy of nucleotide base assignment was checked manually in Geneious 6.1.2. (Biomatters Ltd). Sequence alignment was performed with the Geneious Aligner algorithm under default conditions, and confirmed by eye.

Microsatellite loci developed specifically for *P. glauca* were obtained from Fitzpatrick, Shivji, Chapman, and Prodöhl (2011) and Mendonça et al. (2012), but only twelve loci consistently amplified in samples from Atlantic nursery areas, of which only seven loci were successfully screened in samples from Brazil (Table S1). Polymerase chain reaction amplifications used the forward primer of each locus with an added T3 tail complementary to one of four fluorescently labeled T3 primers (e.g., 6‐FAM, VIC, NED, or PET). Two multiplex reactions (Multiplex I: *Pgla‐05, Pgla‐07, TB04,* tb15; Multiplex II: *Pgla‐03; Pgla‐04; Pgla08; TB01; TB02; TB13;* Table S1) contained 5 μl of *Taq* PCR Master Mix Kit (Qiagen), 3 μl of ultra‐pure water, 1 μl of primer mix (details available from the authors upon request), and 1 μl of gDNA (5–20 ng). All multiplex PCR reactions began with a hot start at 95°C for 15 min followed by: (1) 17 cycles of a denaturation step of 95°C for 30 s, a touchdown annealing step of 90 s and between 62 and 54°C with a 0.5°C decrease per cycle, and an extension step at 72°C for 30 s; (2) 15 cycles of a denaturation step as above, annealing at 54°C for 45 s, and extension as above, and by (3) 8 cycles of a denaturation step as above, annealing at 53°C for 30 s, and extension as above. A final extension step was conducted at 60°C for 30 min. Two loci (*Pgla‐01; Pgla‐10;* Table S1) were amplified separately in 5 μl of *Taq* PCR Master Mix Kit (Qiagen), 3.2 μl of ultra‐pure water, 0.04 μmol/l of T3‐tailed forward primer, 0.4 μmol/l of reverse primer, 0.4 μmol/l of fluorescent dye, and 1 μl of gDNA. Single locus PCR conditions were similar to those for multiplex reactions except that the annealing steps were decreased by 15 sec at the 54 and 53°C cycles of the PCR program. The presence, length, and quality of each amplification product were verified by electrophoresis on a 2% (w/v) agarose gel and diluted 0.8× if needed. One microliter of each diluted PCR product was added to 10 μl of deionized formamide and to 0.2 μl of the internal size standard Genescan‐500 LIZ (Applied Biosystems) and run on an ABI 3130*xl* Genetic Analyzer (Applied Biosystems). Genemapper software 4.1 (Applied Biosystems) was used to manually score individual genotypes.

### 2.2 Genetic diversity analyses

Mitochondrial DNA diversity indices were calculated with DnaSP version 5 (Librado & Rozas, 2009) including the total number of haplotypes (H), haplotype diversity (*h*), and nucleotide diversity (π) per sample collection, and the average number of nucleotide differences among sequences within each sample collection (*k*) and over all sequences. The relationships among haplotypes and their spatial distribution were inferred by constructing a 95% parsimony inference network (Clement, Posada, & Crandall, 2000) as implemented in POPART (Leigh & Bryant, 2015). Bayesian inference (BI) of the phylogenetic relationships among haplotypes was performed with MrBayes 3.2 (Ronquist et al., 2012), using two independent Markov runs with four chains each (using default heating parameters). The model of nucleotide substitution for the mtDNA CR fragment used in MrBayes was estimated in MEGA 5.2.2 (Tamura et al., 2011), based on the corrected Akaike Information Criterion (AICc). Since the best substitution model was TN93 (Tamura & Nei, 1993), which is not available in MrBayes 3.2, we used the next most complex model (and the second best in AICc value) instead, that is, the General Time Reversible model with gamma‐distributed rate variation across sites and a proportion of invariable sites as the model of sequence evolution. The runs included a total of five million generations, discarding the first 25% of generations as burn‐in. Convergence between runs was confirmed by observing a mean standard deviation of split frequencies of <0.01 between runs, as indicated in the software manual, and effective sample sizes >200 for the combined parameter files calculated using Tracer version 1.6 (Rambaut, Suchard, Xie, & Drummond, 2014). Two sets of additional mtDNA CR sequences were obtained from GenBank for *Carcharhinus plumbeus* (Accession no.s AY766129–Ay766136) and for *C. limbatus* (Accession no.s GU245557–GU245566) to serve as outgroups.

Multilocus microsatellite genotypes were checked for stuttering, allele dropout, and the presence of null alleles using MicroChecker version 2.2.3 (van Oosterhout, Hutchinson, Wills, & Shipley, 2004). Genetic relatedness values among all pairs of individuals within and among nursery collections were estimated using the R package *related* (Pew, Muir, Wang, & Frasier, 2015) according to package manual. Four genetic relatedness estimators, namely Li, Weeks, and Chakravarti (1993), Lynch and Ritland (1999), Queller and Goodnight (1989), and Wang (2002), were first evaluated for performance using the function “compareestimators” in the R package *related*. This function generates simulated individuals of known relatedness based on the observed allele frequencies and calculates the genetic relatedness using four different estimators. The correlation between observed and expected genetic relatedness was obtained for each estimator, and the one with the highest r (correlation coefficient) was chosen. Genetic relatedness among all pairs of small juvenile blue sharks were then estimated and visually inspected for the presence of outlier pairs. The distribution of observed pairwise relatedness values across all juvenile blue sharks was also compared to the values expected between parent‐offspring (PO), full‐siblings (FS), half‐siblings (HS), and unrelated pairs (UN). This was meant to check the presence of possibly related individuals as they may bias estimates of genetic diversity and differentiation (e.g., Nielsen et al., 2009). Expected genetic relatedness values were generated for 100 pairs of individuals per degree of relationship (i.e., PO, FS, HS, UN) using the observed allele frequency data. Finally, the average relatedness within and across sample collections was tested for deviations from random mating expectations using 1,000 permutations of multilocus genotypes between collections, while keeping group size constant (i.e., size of sample collection). All computations were performed with the R package *related* (Pew et al., 2015) and following the manual guidelines.

Genetic diversity indices at the nuclear microsatellite loci, including the total number of alleles per locus, the mean number of alleles across loci per collection, allelic richness (*A*_R_; *n* = 37), and observed and expected heterozygosities (*H*_*O*_ and *H*_*E*_, respectively), were estimated in FSTAT version 2.9.3.2 (Goudet, 2002). Genepop on the web version 4.2 (Raymond & Rousset, 1995; Rousset, 2008) was used to test for linkage disequilibrium between each locus within and across sample collections and to test whether the genotypic distributions at each locus across and within sample collections were in accordance with Hardy–Weinberg Equilibrium (HWE) expectations. *P*‐value estimates for both tests were based on 10,000 dememorizations, 100 batches, and 10,000 iterations per batch, and corrected for multiple tests using a False Discovery Rate correction (FDR; Benjamini & Hochberg, 1995).

### 2.3 Genetic differentiation analyses

Levels of genetic differentiation among all sampling collections were estimated in Arlequin 3.5.2 (Excoffier & Lischer, 2010), by means of pairwise Φ_ST_ tests at the mtDNA CR based on combined nucleotide diversity and haplotype frequencies and of pairwise *F*_ST_ tests at the microsatellite loci based on the number of alleles. In addition, an analysis of molecular variance (AMOVA) was performed to test different null hypotheses of genetic structure: (1) genetic homogeneity among nursery collections, at each time period; (2) genetic homogeneity across Atlantic nursery collections between time periods (i.e., 2000 vs. 2010s); and (3) genetic homogeneity between the Brazilian collection and the three Atlantic nurseries. A locus‐by‐locus AMOVA was also performed on the nuclear microsatellite data using Arlequin, to infer the contribution of the different loci to the overall genetic structure. In all cases, statistical significance was assessed by 10 000 permutations of the dataset. As the locus‐by‐locus AMOVA did reveal contrasting signals of genetic differentiation among loci, we performed outlier tests aiming at detecting loci not conforming to neutrality expectations using LosiTan (Antao, Lopes, Lopes, Beja‐Pereira, & Luikart, 2008). This software implements the FDIST approach of Beaumont and Nichols (1996) and was run with default parameters, following the guidelines in Antao et al. (2008).

The statistical power of the microsatellite‐based *F*_ST_ tests (i.e., rejection of the null hypothesis H_0_ of genetic homogeneity when it is false) and the corresponding alpha level (i.e., rejection of H_0_ when it is true) were estimated in POWSIM version 4.1 (Ryman & Palm, 2006). Analyses were conducted using 10,000 burn‐in steps, 100 batches, and 1,000 iterations per batch, for values of *F*_ST_ ranging from 0.0001 to 0.01. The lower *F*_ST_ value tested is the minimum acceptable by POWSIM, but may reflect migration rates ~0.1 among populations (i.e., possibly leading to demographic connectivity; Hastings, 1993) using Takahata's (1983) formula as an approximation of the resulting *F*_ST_ value:

$$F_{\mathrm{ST}}=1/\left[1+4N_{e}m{\left(d/d-1\right)}^{2}\right]$$

assuming a finite island model with *d* demes (with *d *=* *2, for pairwise comparisons), and an *N*_*e*_ estimate obtained as described below. Power analyses were conducted using 50 individuals per deme and allelic frequencies at microsatellite loci.

The genetic structure among blue shark nursery collections was further evaluated by a Discriminant Analysis of Principal Components (DAPC) on the multilocus microsatellite genotype data, following the method of Jombart, Devillard, and Balloux (2010) implemented in R (R Development Core Team, 2016). Prior to running the DAPC, the alpha‐score was first estimated to assess the number of PCs to retain and the ability of the DAPC in discriminating groups. The *K*‐means method was run for *K* = 1–12 (i.e., twice the number of sample collections), and the best *K* value was chosen using the Bayesian Information Criterion (Jombart et al., 2010). The DAPC was run using the most likely *K* value and first two PCs explaining ~80% of the variance, as well as using location information.

Genetic admixture and differentiation among blue shark nursery collections was also evaluated with the clustering method implemented in STRUCTURE (Falush, Stephens, & Pritchard, 2003; Pritchard, Stephens, & Donnelly, 2000) using nuclear microsatellite genotypes. STRUCTURE was run using an admixture ancestry model with correlated allelic frequencies and no prior information of sample location. Five replicates were run for each *K*‐value tested, using 500,000 burn‐in steps followed by 1 million steps. Criteria for choosing the best *K*‐values followed those indicated in the software manual. Given the reduced number of microsatellite loci genotyped for the Brazilian collections, the DAPC and STRUCTURE were run with two datasets: One dataset included only the nursery sample collections making use of the full set of microsatellite markers, and a second dataset included all nursery and the Brazilian sample collections with the reduced set of loci.

### 2.4 Comparison between Atlantic and Indo‐Pacific blue sharks

Atlantic and Indo‐Pacific blue shark genetic diversity was compared using mtDNA CR sequences. Indo‐Pacific sequence data were obtained from GenBank based on previous work by Ovenden et al. (2009) including samples from Indonesia (*n* = 19), Japan (*n* = 20), west Australia (*n* = 4), and east Australia (*n* = 17). Briefly, the nucleotide sequence for haplotype PG01 (Ovenden et al., 2009) was obtained from GenBank Accession no. FJ161689, with the remaining haplotypes and haplotype frequencies being reconstructed according to Table 7 in Ovenden et al. (2009). Given the absence of genetic differences among the four Indo‐Pacific collections, all sequences were included in a single group (hereon referred to as IP) in all downstream analyses. The final Atlantic mtDNA CR alignment was trimmed to a homologous fragment of Indo‐Pacific sequences of 373 bp in length. Pairwise Φ_ST_ tests were performed in Arlequin, and a haplotype network of Atlantic versus Indo‐Pacific sequences was also obtained in POPART, as described above.

### 2.5 Demographic analyses

Contemporary effective population size was estimated for the whole Atlantic population, using the temporal method of Jorde and Ryman (1995) as implemented in NeEstimator version 2.01 (Do et al., 2014). To this end, we used the two temporal groups as input populations and considered a generation time lapse of 2 between groups, since the age at maturity in female *P. glauca* is ~ 4–5 years (Skomal & Natanson, 2002) and the modal cohort in each temporal group is 2005 and 2013 for the 2000 and 2010s groups, respectively (Figure S1).

Past changes in population size were investigated using mtDNA CR sequences, by means of Fu's *F*_s_ neutrality test (Fu, 1997) using 10,000 simulations of random data and an α = 0.05, as implemented in Arlequin version 3.5 (Excoffier & Lischer, 2010). Microsatellite genotypes were also used to test for recent bottleneck events within Atlantic waters (but excluding the Brazilian collection) using the excess heterozygosity test implemented in Bottleneck version 1.2.02 (Piry, Luikart, & Cornuet, 1990). The discrepancy between heterozygosity values was tested using a Wilcoxon's signed‐rank test under the null hypothesis of no significant heterozygosity excess. Given the importance of mutation model specification in bottleneck detection (Peery et al., 2012), we ran bottleneck using the two‐phase model as the most likely model of microsatellite mutation (Di Rienzo et al., 1994), and including all combinations of three probabilities of single step mutations (70%, 80%, and 90%) and three values of variance among multiple steps (3, 12, and 30), for 1,000 replicates each. The above values were chosen based on the findings and recommendations in Peery et al. (2012).

## 3 Results

### 3.1 Genetic diversity in Atlantic blue sharks

A total of 237 individuals from the three Atlantic blue shark nurseries and from off Brazil were successfully sequenced for a 899‐bp fragment of mtDNA CR. The final alignment included two indels and 29 polymorphic sites, of which 19 were transitions and 11 were transversions. Fifty‐two haplotypes were recovered from the final alignment (after removal of indels; GenBank Accession no.s KY923141‐KY923192) showing an overall mean *k* of 3.45, and overall π and *h* of 0.0038 and 0.90, respectively. The levels of genetic diversity in the mtDNA CR were very similar among sample collections (Table 1), showing high haplotype diversities (*h*: 0.88–0.95) and equally differentiated haplotypes (*k*: 3.00–3.57).

**Figure 2:**
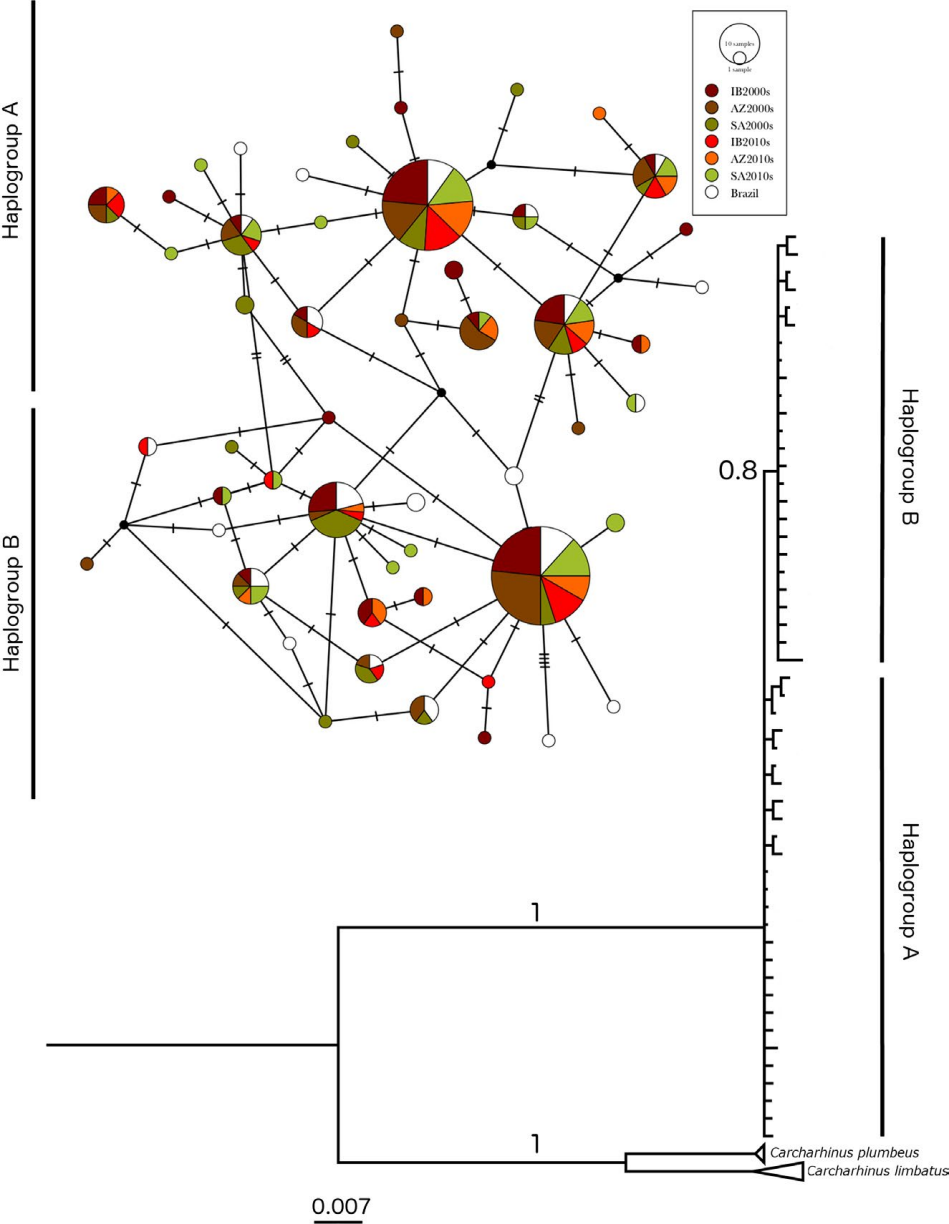
Mitochondrial control region haplotype network and Bayesian inference (BI) tree based on Atlantic blue shark sample collections. Number of mutated positions among connected haplotypes in the network are indicated by the slash marks. Numbers on the tree branches are BI probabilities. Black circles indicate inferred (i.e., not observed) haplotypes

The haplotype network showed two groups of haplotypes (or haplogroups) separated by two‐step mutations, with the most frequent haplotypes in each group being connected by a minimum of four mutation steps and by two low‐frequency haplotypes (Figure 2). Each haplogroup shows a star‐shaped structure, with haplogroup B also showing much reticulation among haplotypes. No evidence of differential spatial distribution of haplotypes among sample collections is apparent from the haplotype network, with most shared haplotypes having nearly equal frequencies in each nursery and in Brazil (Figure 2). Pairwise comparison of all mtDNA CR sequences shows a unimodal distribution with a mode at 4 bp, while haplogroups A and B each have unimodal distributions at 2 bp and 1 bp, respectively (Figure S2). Bayesian reconstruction of phylogenetic relationships among *P. glauca* haplotypes confirmed the existence of the two haplogroups (with moderate support) and showed haplogroup B nested within haplogroup A. The two haplogroups have equal relative frequencies in all nursery sampling collections, although haplogroup A is more frequent in the Brazilian collection (Table S2).

Twelve microsatellite loci were genotyped for 302 juvenile blue sharks from the three Atlantic nursery areas (i.e., excluding Brazil), showing a total number of alleles per locus ranging from 2 to 35 (mean: 10.6) and *H*_*O*_ ranging from 0.20 to 0.93 (mean: 0.60; Table S1). No evidence of stuttering or allele scoring errors was found, but null alleles were detected at loci *Pgla03* for SA2000 and AZ2010, *Pgla05* for AZ2000, *Pgla07* for all collections except AZ2000, *Pgla10* for AZ2000, and *TB15* for IB2010. The genetic relatedness estimates of Li et al. (1993) showed the best performance with our data (*r *=* *0.796), demonstrating that small juvenile blue sharks across all nursery collections followed closely the distribution of values expected from unrelated pairs of individuals (Figure S3). Also, observed average relatedness per collection did not deviate from expectations under random mating within and across nurseries (*p* > .05). Thus, all individuals collected at nursery areas were considered unrelated and included in further genetic analyses.

The levels of genetic diversity at the nuclear microsatellite loci were very similar among all nursery sample collections, with mean *A*_R_ between 7.15 and 7.96 and mean *H*_*O*_ of 0.61 and 0.65 (Table 1). Concordantly, no significant temporal difference in *H*_*O*_, number of alleles or *A*_R_ was found across Atlantic nurseries (*p *>* *0.05) despite the two North Atlantic nurseries showing lower values in 2,010. When considering the reduced set of microsatellite loci and the Brazilian collection, similar levels of genetic diversity were also found among all collections (Table S3). Linkage disequilibrium tests detected no significant associations either within or between sample collections. Loci *Pgla*03 for SA2000s and *Pgla*05 for IB2000s deviated from HWE expectations (after FDR correction), both due to heterozygote deficits. Locus *Pgla07* did not conform to HWE expectations across all nursery samples or in the Brazil sample (after FDR correction), also due to heterozygote deficit, and was removed from further analyses.

### 3.2 Genetic differentiation among Atlantic collections of blue sharks

Pairwise Φ_ST_ tests based on mtDNA haplotype frequency data showed no significant genetic differentiation among sample collections, with comparisons including Brazil showing larger Φ_ST_ values (Table 2). Concordantly, pairwise *F*_ST_ tests based on microsatellite genotypes also showed no significant differentiation among nursery collections. Comparisons including the Brazilian sample showed larger pairwise *F*_ST_ values, with one comparison (Brazil vs. IB2000) being significant (Table 2).

**Table 2:** Pairwise *F*_ST_ (below diagonal) based on 11 nuclear microsatellite loci, and Φ_ST_ values (above diagonal) based on the mtDNA CR

	IB2000s	AZ2000s	SA2000s	IB2010s	AZ2010s	SA2010s	Brazil
IB2000s		−.007	−.005	−.019	−.010	−.015	.006
AZ2000s	−.002		.010	−.015	−.004	−.009	.011
SA2000s	.000	.003		−.014	.006	−.018	.001
IB2010s	.000	−.003	.004		−.011	−.024	−.001
AZ2010s	.000	.001	.009	−.002		−.010	.025
SA2010s	−.001	−.001	.004	−.002	.001		.004
Brazil^a^	**.013**	.007	.014	.007	.013	.007	

a Values based on six loci, as indicated in text. Significant *P*‐value (after FDR correction) in bold.

Power simulations indicated our microsatellite panel had high power (>95%) to detect pairwise *F*_ST_ values >0.005 but low power to detect values in the range of 0.0025–0.0001 (<60% and <5%, respectively), for an α value of 0.04, regardless of the number of loci used (i.e., 11 or six) or sampling scheme tested (i.e., including only Atlantic nursery collections, and including Atlantic nursery and Brazilian collections, respectively). For the lower *F*_ST_ value and 11 loci, increasing the sample size per deme from 50 to 500 individuals still showed <20% power to reject the null hypothesis.

Results of the AMOVA confirmed the lack of significant genetic heterogeneity among nursery collections at the mtDNA CR or at the microsatellite loci, regardless of the time period considered. Likewise, no significant temporal differences were detected when considering the three nursery collections together per time period (i.e., 2000s vs. 2010s). However, the AMOVA showed that samples from Atlantic nursery areas were weakly but significantly differentiated from the Brazilian collection both at the mtDNA CR and at the nuclear microsatellite loci (Table 3). However, in the locus‐by‐locus AMOVAs based on microsatellite data, locus *Pgla03* showed *F*_ST_ and *F*_CT_ values about 1–2 orders of magnitude higher than the remainder loci. Removal of this locus from the analyses yielded nonsignificant *F*_CT_ values (data not shown). Despite these results, all microsatellite loci were found to conform to neutrality expectations, both when considering only the samples from nursery areas genotyped at 11 microsatellite loci (*n* = 6 “populations”), and when considering all Atlantic collections genotyped at only six loci (*n* = 7 “populations”).

**Table 3:** Analysis of molecular variance among Atlantic collections of *P. glauca*

	Nuclear microsatellites			mtDNA CR		
	*F*_ST_	*F*_SC_	*F*_CT_	*F*_ST_	*F*_SC_	*F*_CT_
H0: Panmixia among Atlantic Nurseries						
2000s	−0.0003			−0.002		
2010s	−0.0005			−0.015		
H0: Temporal differences across Atlantic nurseries						
2000s vs. 2010s	−0.0002	−0.0004	0.00019	−0.011	−0.007	−0.004
H0: Atlantic Nurseries vs. Brazil						
2000s vs. Brazil^a^	0.010*	−0.002	0.013**	0.007	−0.002	0.008**
2010s vs. Brazil^a^	0.008*	0.000	0.008**	0.009	−0.015	0.023**

^a^Analyses based on nuclear microsatellite loci and including Brazil are based on only six loci, as indicated in the text.

Significant *P*‐values are *.05; **.005.

**Figure 3:**
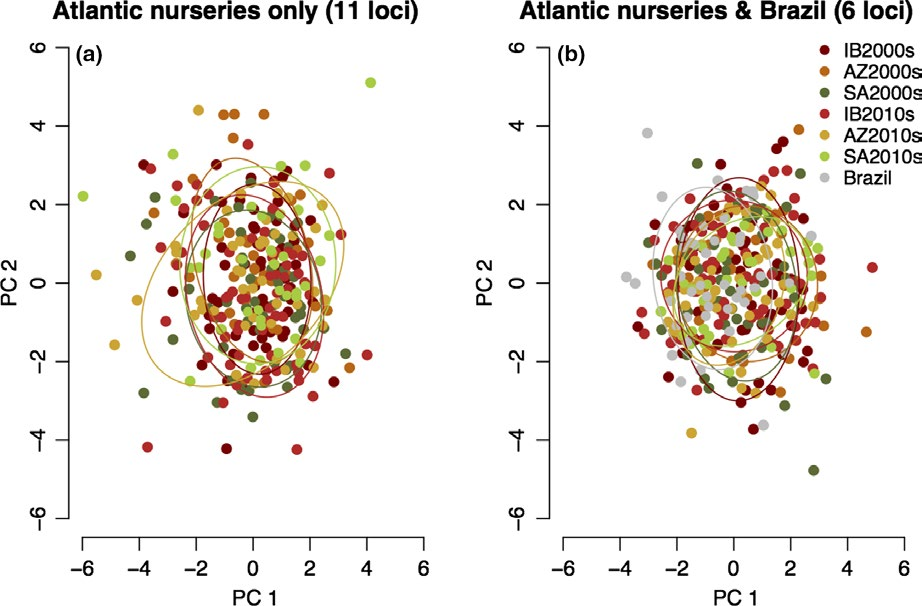
Discriminant analysis of principal components of (a) 11 microsatellite loci genotyped at the three Atlantic nursery sample collections, and (b) six microsatellite loci genotyped at the three nursery areas and the Brazil sample collections

The DAPC had little discriminating power among Atlantic nursery collections, or among nursery collections and Brazil, as indicated by the very low alpha values (<0.1; Figure S4). This was evident in the final DAPC using either the *K*‐means clustering method (data not shown) or location information (Figure 3), where samples from different clusters/collections had largely overlapping clouds of points. Consistent with these findings, the STRUCTURE runs showed that the most likely *K* value was 1 in the two datasets considered (Figure S5), with individuals having equal probabilities of membership across clusters regardless of the *K* value (data not shown).

### 3.3 Comparison between Atlantic and Indo‐Pacific blue sharks

The final alignment of homologous mtDNA CR sequences spanning 373 bp in length produced 41 haplotypes, of which 11 were shared between Atlantic and IP samples (Table S4). The resultant haplotype network showed two groups of haplotypes differing in two step mutations, as seen for Atlantic‐only samples (Figure S6). Many of the haplotypes shared between Atlantic and IP samples coincided with the most frequent haplotypes in each haplogroup, with haplogroup frequencies in the overall Atlantic and IP samples approaching 50:50 (Table S4). Pairwise Φ_ST_ tests between the Atlantic (three groups of samples, i.e., 2,000s, 2,010s, and Brazil) and IP collections indicated low and nonsignificant differences in all comparisons (Φ_ST_: 0–0.009, *P *>* *0.05).

### 3.4 Demography of Atlantic blue sharks

The estimated contemporary effective size of the Atlantic blue shark population was 4,513 (parametric bootstrap 95% confidence interval: 3,422–5,752). Neutrality tests based on mtDNA CR sequences and Fu's *F*_S_ test detected significant deviations from selective neutrality and population equilibrium in Atlantic nurseries (considered together per time period) and in the Brazilian collection. In all cases, significantly large negative Fu's *F*_s_ values were found (2000s group: *F*_s_ = −18.46; 2010s group: *F*_s_ = −26.06; Brazil: *F*_s_ = −14.42; all *P*‐values < 0.001), suggesting past population expansions. When considering the two mtDNA CR haplogroups detected above, both haplogroups also had significant negative Fu's *F*_S_ values (A: −16.93; B: −17.12; *P *<* *0.000), consistent with past population size expansion. On the other hand, analysis of historical population size changes using nuclear microsatellite loci found no signal of a recent population bottleneck, regardless of the parameter combination used (*P *>* *0.05).

## 4 Discussion

### 4.1 Blue shark genetic diversity and population structure

Genetic homogeneity at the Atlantic‐wide level was found for the blue shark in the present study. Juvenile sharks born at three distinct Atlantic nursery areas, located on either side of the Equator, share the same genetic composition at 11 nuclear microsatellites and at the mtDNA CR (Tables 1 and 2). The lack of genetic structure among Atlantic nursery areas was consistently found in the two temporal sampling replicates. Moreover, genetic homogeneity at the maternally inherited mtDNA CR indicates that adult female blue sharks may give birth in nursery areas other than their natal ones, even if located in opposite hemispheres. Thus, there is no evidence of female phylopatry to discrete Atlantic nursery areas in *P. glauca*.

The genetic homogeneity among Atlantic nursery areas and across hemispheres is in agreement with previously published genetic studies on the population genetics of blue sharks. Specifically, genetic homogeneity at mitochondrial and nuclear markers was found in blue sharks sampled at various locations within the North Atlantic (Queiroz, 2010), and within the North Pacific (King et al., 2015), as well as across the Atlantic and the Pacific basins (this study; Ovenden et al., 2009; Taguchi et al., 2015). The very low or zero genetic differentiation observed across such large spatial scales suggests that enough gene flow currently exists among distant areas resulting in homogenous allelic frequencies, or that it occurred until very recently and genetic drift has not yet had time to produce detectable differences among areas. Regardless, all studies so far consistently indicate that blue sharks exhibit/exhibited unrestricted gene flow at the within‐ocean basin level mediated both by females and by males.

The Brazilian blue shark samples show some discordance with the above pattern of unrestricted gene flow at large spatial scales. Low but significant genetic differentiation was found between the Atlantic nurseries and Brazil at both nuclear and mtDNA loci, albeit with a stronger signal at the microsatellite loci. This result is surprising given the absence of genetic differences among Atlantic collections on either side of the Equator, and the potential for long distance dispersal and apparent lack of barriers for the blue sharks throughout the Atlantic and Pacific oceans (this study; Ovenden et al., 2009; King et al., 2015; Taguchi et al., 2015). Given the low gDNA quality of the Brazilian samples and its effect in amplification and scoring of half of the microsatellite loci, allele scoring in the six loci successfully amplified may have been affected also, falsely indicating genetic differentiation to exist. This is of particular notice as genetic differentiation at nuclear microsatellites is strongly influenced by a single locus (i.e., *Pgla03*), which also showed evidence of null alleles at two sample collections (SA2000s and AZ2000s) and significant deviations from HWE expectations (in SA2000s). Future confirmation of this signal is thus needed, and we caution against definitive conclusions regarding the existence of a discrete population of blue sharks in the western South Atlantic.

The current study also shows evidence of high genetic connectivity between Atlantic and Indo‐Pacific blue sharks at the mtDNA CR. The most common haplotypes within each region were shared, and there were no observed phylogeographic breaks among ocean basins in *P. glauca*. Moreover, the levels of genetic differentiation between basins were very low and not significant. These results suggest that gene flow is occurring within as well as among whole ocean basins. Connectivity between the South Atlantic and the western Indian oceans has been proposed by da Silva et al. (2010) based on the length frequency distributions of blue shark longline catches and tag‐recapture data. These authors argued that blue sharks around South African waters may comprise a population spanning the South Atlantic and Indian Ocean, possibly extending throughout the Southern Hemisphere (da Silva et al., 2010). The absence of female blue shark phylopatry to discrete pupping areas, coupled to the species' high dispersal potential and wide thermal range, may effectively allow unrestricted dispersal throughout the world's oceans.

In contrast to the above finding, previous studies on blue shark biology in Atlantic and Pacific waters have pointed out differences in key life‐history parameters (e.g., size and age at maturity, and growth rates) within as well as between ocean basins (e.g., Pratt, 1979; Castro & Mejuto, 1995; Skomal & Natanson, 2002; Lessa, Santana, & Hazin, 2004; Francis & Duffy, 2005; Nakano & Stevens, 2008 and references therein; Jolly et al., 2013), suggesting population differentiation. However, many of these studies suffer from inadequate, incomplete, and biased samples sizes (Skomal & Natanson, 2002; Tanaka, Cailliet, & Yudin, 1990), such as the absence of a representative size range in specimens, which compromises both the accuracy of the parameter estimates and interstudy comparisons. Thus, further studies are still needed to reliably test for spatial differences in key life‐history traits.

Available literature on the population genetic structure of other oceanic epipelagic sharks (*sensu* Stevens, 2010) indicates that connectivity between Atlantic and Indian/Indo‐Pacific basins was found also in the crocodile shark *Pseudocarcharias kamoharai* (Matsubara, 1936) (da Silva Ferrette et al., 2015) and the basking shark *Cetorhinus maximus* (Gunnerus, 1765) (Hoelzel, Shivji, Magnussen, & Francis, 2006), and two other species showed genetic homogeneity at the within‐ocean basin level, *that is,* the whale shark *Rhincodon typus* Smith, 1828 (Castro et al., 2007; Schmidt et al., 2007; Vignaud et al., 2014) and the bigeye tresher *Alopias superciliosus* Lowe, 1841 (Morales, 2012). However, most species of large oceanic epipelagic sharks show genetic structure at smaller spatial scales than expected based on their high dispersal ability, such as the pelagic thresher shark *Alopias pelagicus* Nakamura, 1935 (Cardeñosa, Hyde, & Caballero, 2014), the great white *Carcharodon carcharias* (Linnaeus, 1758) (Andreotti et al., 2016; Blower, Pandolfi, Bruce, Gomez‐Cabrera, & Ovenden, 2012; Jorgensen et al., 2010; O' Leary et al., 2015), the mako shark *Isurus oxyrinchus* Rafinesque, 1810 (Heist, Musick & Graves, 1996; Schrey & Heist, 2003), the silky shark *Carcharhinus falciformis* (Müller & Henle, 1839) (Clarke et al., 2015; Galván‐Tirado, Díaz‐Jaimes, García‐de León, Galván‐Magana, & Uribe‐Alcocer, 2013), and the oceanic whitetip *Carcharhinus longimanus* (Poey, 1861) (Camargo et al., 2016). Clearly, the combination of pelagic habit, oceanic habitat, and wide distribution ranges does not necessarily result in widespread mixing of individuals across large spatial scales. Other factors need to be taken into account when drawing inferences on putative population structure patterns of this group of species, such as site fidelity to mating/pupping grounds (Andreotti et al., 2016; Blower et al., 2012; Cardeñosa et al., 2014; Jorgensen et al., 2010; O' Leary et al., 2015; Schrey & Heist, 2003) or, simply, lower than expected dispersal ability (Camargo et al., 2016).

Current distribution patterns of genetic diversity are also influenced by a variety of factors including historical demographic events (e.g., population size changes), vicariant/dispersal events, selection, etc., which may be shaped by both biological and environmental processes. Environmental variability associated with the glacial‐interglacial cycles of the Late Quaternary predating the appearance of modern climatic conditions (~11.5 ky before present) have been recurrently invoked to explain present‐day phylogeographic patterns in a variety of living organisms (e.g., Maggs et al., 2008; Randi, 2007). For instance, colder water conditions around the tip of South Africa during glacial periods, and the presence of the cold Benguela current during warm interglacial periods have been proposed to explain Atlantic versus Indo‐Pacific isolation and mitochondrial lineage divergence in warm‐temperate and tropical sharks, such as in cosmopolitan oceanic epipelagic species (e.g., *R. typus,* Vignaud et al., 2014; *C. falciformis,* Clarke et al., 2015) and in many cosmopolitan coastal pelagic carcharhinoids (e.g., *C. limbatus,* Keeney & Heist, 2006; Keeney, Heupel, Hueter, & Heist, 2005; *Carcharhinus obscurus* (Lesueur, 1818) Benavides et al., 2011; *Carcharhinus plumbeus* (Nardo, 1827)*,* Portnoy, McDowell, Heist, Musick, & Graves, 2010; *Carcharhinus brachyurus* (Günther, 1870), Benavides et al., 2011; Benavides et al., 2011; *Galeocerdo cuvier* (Péron & Lesuer, 1822) Bernard et al., 2016; *Sphyrna lewini* (Griffith & Smith, 1834), Duncan, Martin, Bowen, & De Couet, 2006; Daly‐Engel et al., 2012). Lineage divergence associated with between‐ocean cessation of gene flow in temperate species with wide temperature ranges, such as the blue shark, may also have occurred during periods when water temperatures around South Africa went below their thermal tolerance limit. However, extensive interbasin gene flow resuming during interglacial periods (as seen presently) may homogenize the distribution of mtDNA lineages globally.

### 4.2 Demography of blue sharks

Effective population size estimates for Atlantic and Pacific blue sharks are concordant with a contemporary *N*_*e*_ of ~ 4,000–5,000 (this study, King et al., 2015). The similar *N*_*e*_ estimates were obtained despite the different sample sizes of the two studies (302 vs. 844, respectively), and the distinct estimation methods used (temporal method of Jorde & Ryman, 1995 vs. linkage disequilibrium method implemented in NEEstimator, Do et al., 2014). This convergence of independent estimates suggests relatively accurate contemporary *N*_*e*_ values (Hare et al. 2011). If genetic panmixia occurs between Atlantic and IP blue sharks, then it is perhaps expected that *N*_*e*_ should be similar throughout the species range and thus reflect global contemporary *N*_*e*_ values.

In any case, populations with contemporary *N*_*e*_ values >3,000 have been suggested to be at a lower risk of loss of genetic diversity even when subjected to high fishing mortality (~90% bottleneck; Pinsky & Palumbi, 2014). Indeed, we found no evidence of recent population bottlenecks in Atlantic blue sharks, and King et al. (2015) also found concordant estimates of contemporary and historical *N*_*e*_ values in the Pacific Ocean, suggesting little population size variation in the past. In turn, evidence of past population expansion was found at the mtDNA level in both Atlantic and Pacific samples (this study, Taguchi et al., 2015).

Surprisingly, *N*_*e*_ estimates for blue sharks appear somewhat low given the very high abundance of the species worldwide, with annual estimates of ~10^7^ individuals being traded in the global fin market alone (Clarke, 2008; Clarke et al., 2006). Assuming a census size ~10^8^ globally, the ratio of contemporary *N*_*e*_ to census size (*N*_*c*_) of blue sharks may be in the order of 10^‐5^. The *N*_*e*_/*N*_*c*_ ratio of blue sharks appears to be much smaller than the one observed in another pelagic carcharhinid, the sandbar shark (*N*_*e*_/*N*_*e*_~0.5; Portnoy, McDowell, McCandless, Musick & Graves, 2009). Theoretical expectations based on generation time, age at maturity (α), and adult life span (AL) predict that in species with larger AL/α (i.e., where lifetime reproductive output is higher), the *N*_*e*_/*N*_*c*_ will be lower (Waples, Luikart, Faulkner, & Tallmon, 2013). Thus, the much lower *N*_*e*_/*N*_*c*_ in blue sharks compared to sandbar sharks are in agreement with these expectations, given its much shorter generation times (4–5 vs. 15–16 year) and longer AL (11 vs. 5 year).

### 4.3 Management considerations

The genetic homogeneity across whole ocean basins seen in Atlantic (present study) and Pacific oceans (Ovenden et al., 2009; Taguchi et al., 2015) is at odds with the currently assumed distinction of northern and southern stocks within each ocean basin. Indeed, the bulk of the evidence gathered thus far indicates that the blue shark exhibits dispersal with gene flow over very large spatial scales, and little to no phylopatry to the sampled nursery areas or to distinct ocean basins. Nevertheless, ocean‐wide genetic stocks (i.e., where allele frequencies are similar among distant locations) can be maintained at the rate of only a few long‐distance migrants per generation and thus does not preclude the existence of several demographic stocks relevant to stock assessment and management (Palsbøll, Berube, & Allendorf, 2007). Levels of gene flow among areas are often difficult to estimate when *F*_ST_ values are very small, as the relationship between *F*_ST_ and m*N*_*e*_ (i.e., the effective number of migrants per generation) has a long flat right hand tail as *F*_ST_ approaches zero and *N*_*e*_ > 1,000s (Lowe & Allendorf, 2010; Waples, 1998). Indeed, genetic connectivity may be reached at migration rates much lower than those leading to demographic connectivity among areas (Lowe & Allendorf, 2010; Ovenden, 2013; Palsbøll et al., 2007). However, in cases where effective populations sizes are ~1,000s, as in blue sharks, the levels of genetic divergence associated with migration rates which could lead to demographic connectivity (~10%; Hastings, 1993) may be difficult to detect using traditional molecular markers and moderate to large samples sizes (~50–500 individuals per population; e.g., White, Fotherby, Stephens, & Hoelzel, 2010; this study).

Based on the above, the precautionary approach in conservation and fisheries management would be to consider each nursery area as independent, with potentially different demographic parameters and vulnerability to fishing pressure. If each nursery area currently exchanges only a few migrant individuals per generation with other nurseries, the replenishment of each stock would be mostly dependent on recruit survival rather than on immigration from adjacent stocks. However, assuming such a scenario would make stock assessment and resource management particularly challenging given the highly migratory nature and complex movement dynamics of blue sharks in time and space (Kohler & Turner, 2008; Nakano & Seki, 2003; Nakano & Stevens, 2008; Queiroz et al., 2012, 2016). Thus, perhaps reconsidering the current assumptions of northern and southern stocks should await additional information on demographic connectivity among nursery areas (e.g., from long‐term tracking studies).

The small sample size and limited geographic coverage of the IP collection used here preclude a definitive conclusion concerning the levels of genetic connectivity of blue shark populations at a global scale; more comprehensive sampling is needed to properly address this issue. However, our results, and those of others (e.g., da Silva et al., 2010), strongly suggest that the South Atlantic “stock” of blue sharks is continuous with (at least) that of the adjacent western Indian Ocean and should thus be managed in concert by the two regional fisheries management organizations implicated, that is, ICCAT and the Indian Ocean Tuna Commission.

## Author Contributions

AV and JRM planned, supervised, and coordinated the work. NQ, GMS, and CdS collected samples (tissue and biological information). IS, AV, and JRM collected, analyzed, and interpreted the data. AV led the writing of the text, with contributions and critical revision by JRM, IS, CdS, CSJ, LRN, GM.

## References

[ece32987-bib-0001] Aires‐da‐Silva, A. M. , Ferreira, R. L. , & Pereira, J. G. (2008). Case study: Blue shark catch‐rate patterns from the Portuguese swordfish longline fishery in the Azores In Camhi M. D., Pikitch E. K., & Babcock E. A. (Eds.), Sharks of the open ocean: biology, fisheries and conservation (pp. 230–235). Oxford, UK: Blackwell Publishing.

[ece32987-bib-0002] Aires‐da‐Silva, A. M. , Hoey, J. J. , & Gallucci, V. F. (2008). A historical index of abundance for the blue shark (*Prionace glauca*) in the western North Atlantic. Fisheries Research, 92, 41–52.

[ece32987-bib-0003] Andreotti, S. , Heyden, S. , Henriques, R. , Rutzen, M. , Meÿer, M. , Oosthuizen, H. , & Matthee, C. A. (2016). New insights into the evolutionary history of white sharks, *Carcharodon carcharias* . Journal of Biogeography, 43, 328–339.

[ece32987-bib-0004] Antao, T. , Lopes, A. , Lopes, R. J. , Beja‐Pereira, A. , & Luikart, G. (2008). LOSITAN: A workbench to detect molecular adaptation based on a F ST‐outlier method. BMC Bioinformatics, 28, 1. 10.1186/1471-2105-9-323 PMC2515854 18662398

[ece32987-bib-0005] Beaumont, M. A. , & Nichols, R. A. (1996). Evaluating loci for use in the genetic analysis of population structure. Proceedings of the Royal Society of London B: Biological Sciences, 263, 1619–1626.

[ece32987-bib-0006] Benavides, M. T. , Feldheim, K. A. , Duffy, C. A. , Wintner, S. , Braccini, J. M. , Boomer, J. , … Cartamil, D. P. (2011). Phylogeography of the copper shark (*Carcharhinus brachyurus*) in the southern hemisphere: Implications for the conservation of a coastal apex predator. Marine and Freshwater Research, 62, 861–869.

[ece32987-bib-0007] Benavides, M. T. , Horn, R. L. , Feldheim, K. A. , Shivji, M. S. , Clarke, S. C. , Wintner, S. , … Chapman, D. D. (2011). Global phylogeography of the dusky shark *Carcharhinus obscurus*: Implications for fisheries management and monitoring the shark fin trade. Endangered Species Research, 14, 13–22.

[ece32987-bib-0008] Benjamini, Y. , & Hochberg, Y. (1995). Controlling the false discovery rate: A practical and powerful approach to multiple testing. Journal of the Royal Statistical Society Series B (Methodological), 57, 289–300.

[ece32987-bib-0009] Bernard, A. M. , Feldheim, K. A. , Heithaus, M. R. , Wintner, S. P. , Wetherbee, B. M. , & Shivji, M. S. (2016). Global population genetic dynamics of a highly migratory, apex predator shark. Molecular Ecology, 25, 5312–5329. 27662523 10.1111/mec.13845

[ece32987-bib-0010] Blower, D. C. , Pandolfi, J. M. , Bruce, B. D. , Gomez‐Cabrera, M. D. , & Ovenden, J. R. (2012). Population genetics of Australian white sharks reveals fine‐scale spatial structure, transoceanic dispersal events and low effective population sizes. Marine Ecology Progress Series, 455, 229–244.

[ece32987-bib-0011] Camargo, S. M. , Coelho, R. , Chapman, D. , Howey‐Jordan, L. , Brooks, E. J. , Fernando, D. , … Foresti, F. (2016). Structure and genetic variability of the oceanic Whitetip Shark, *Carcharhinus longimanus* . Determined Using Mitochondrial DNA. PloS one, 11, e0155623. 27187497 10.1371/journal.pone.0155623 PMC4871334

[ece32987-bib-0012] Camhi, M. D. , Lauck, E. , Pikitch, E. K. , & Babcock, E. A. (2008). A Global Overview of Commercial Fisheries for Open Ocean Sharks In Camhi M. D., Pikitch E. K., & Babcock E. A. (Eds.), Sharks of the open ocean: Biology, fisheries and conservation (pp. 166–192). Oxford, UK: Blackwell Publishing.

[ece32987-bib-0013] Campana, S. E. (2016). Transboundary movements, unmonitored fishing mortality, and ineffective international fisheries management pose risks for pelagic sharks in the Northwest Atlantic. Canadian Journal of Fisheries and Aquatic Sciences, 73, 1599–1607.

[ece32987-bib-0014] Cardeñosa, D. , Hyde, J. , & Caballero, S. (2014). Genetic diversity and population structure of the pelagic thresher shark (*Alopias pelagicus*) in the Pacific Ocean: Evidence for two evolutionarily significant units. PLoS ONE, 9, e110193. 25337814 10.1371/journal.pone.0110193 PMC4206417

[ece32987-bib-0015] Castro, J. A. , & Mejuto, J. (1995). Reproductive parameters of Blue Shark *Prionace glauca*, and other sharks in the Gulf of Guinea. Marine and Freshwater Research, 46, 967–973.

[ece32987-bib-0016] Castro, A. L. , Stewart, B. S. , Wilson, S. G. , Hueter, R. E. , Meekan, M. G. , Motta, P. J. , … Karl, S. A. (2007). Population genetic structure of Earth's largest fish, the whale shark (*Rhincodon typus*). Molecular Ecology, 16, 5183–5192. 18092992 10.1111/j.1365-294X.2007.03597.x

[ece32987-bib-0017] Clarke, S. (2008). Use of shark fin trade data to estimate historic total shark removals in the Atlantic Ocean. Aquatic Living Resources, 21, 373–381.

[ece32987-bib-0018] Clarke, C. R. , Karl, S. A. , Horn, R. L. , Bernard, A. M. , Lea, J. S. , Hazin, F. H. , … Shivji, M. S. (2015). Global mitochondrial DNA phylogeography and population structure of the silky shark, *Carcharhinus falciformis* . Marine Biology, 162, 945–955.

[ece32987-bib-0019] Clarke, S. C. , McAllister, M. K. , Milner‐Gulland, E. J. , Kirkwood, G. P. , Michielsens, C. G. , Agnew, D. J. , … Shivji, M. S. (2006). Global estimates of shark catches using trade records from commercial catches. Ecology Letters, 9, 1125–1126. 10.1111/j.1461-0248.2006.00968.x 16972875

[ece32987-bib-0020] Clement, M. , Posada, D. C. , & Crandall, K. A. (2000). TCS: A computer program to estimate gene genealogies. Molecular Ecology, 9, 1657–1659. 11050560 10.1046/j.1365-294x.2000.01020.x

[ece32987-bib-0021] Cortés, E. , Arocha, F. , Beerkircher, L. , Carvalho, F. , Domingo, A. , Heupel, M. , … Simpfendorfer, C. (2010). Ecological risk assessment of pelagic sharks caught in Atlantic pelagic longline fisheries. Aquatic Living Resources, 23, 25–34.

[ece32987-bib-0022] Daly‐Engel, T. S. , Seraphin, K. D. , Holland, K. N. , Coffey, J. P. , Nance, H. A. , Toonen, R. J. , & Bowen, B. W. (2012). Global phylogeography with mixed‐marker analysis reveals male‐mediated dispersal in the endangered scalloped hammerhead shark (*Sphyrna lewini*). PLoS ONE, 7, e29986. 22253848 10.1371/journal.pone.0029986 PMC3254628

[ece32987-bib-0023] Di Rienzo, A. , Peterson, A. C. , Garza, J. C. , Valdes, A.‐M. , Slatkin, M. , & Freimer, N. B. (1994). Mutational processes of simple sequence repeat loci in human populations. Proceedings of the National Academy of Sciences, 91, 3166–3170. 10.1073/pnas.91.8.3166 PMC43536 8159720

[ece32987-bib-0024] Do, C. , Waples, R. S. , Peel, D. , Macbeth, G. M. , Tillett, B. J. , & Ovenden, J. R. (2014). NeEstimator v2: Re‐implementation of software for the estimation of contemporary effective population size (Ne) from genetic data. Molecular Ecology Resources, 14, 209–214. 23992227 10.1111/1755-0998.12157

[ece32987-bib-0025] Duncan, K. M. , Martin, A. P. , Bowen, B. W. , & De Couet, H. G. (2006). Global phylogeography of the scalloped hammerhead shark (*Sphyrna lewini*). Molecular Ecology, 15, 2239–2251. 16780437 10.1111/j.1365-294X.2006.02933.x

[ece32987-bib-0026] Excoffier, L. , & Lischer, H. E. L. (2010). Arlequin suite ver 3.5: A new series of programs to perform population genetics analyses under Linux and Windows. Molecular Ecology Resources, 10, 564–567. 21565059 10.1111/j.1755-0998.2010.02847.x

[ece32987-bib-0027] Falush, D. , Stephens, M. , & Pritchard, J. K. (2003). Inference of population structure using multilocus genotype data: Linked loci and correlated allele frequencies. Genetics, 164, 1567–1587. 12930761 10.1093/genetics/164.4.1567 PMC1462648

[ece32987-bib-0028] Fitzpatrick, S. , Shivji, M. S. , Chapman, D. D. , & Prodöhl, P. A. (2011). Development and characterization of 10 polymorphic microsatellite loci for the blue shark, *Prionace glauca*, and their cross shark‐species amplification. Conservation Genetics Resources, 3, 523–527.

[ece32987-bib-0029] Francis, M. P. , & Duffy, C. (2005). Length at maturity in three pelagic sharks (*Lamna nasus*,* Isurus oxyrinchus*, and *Prionace glauca*) from New Zealand. Fishery Bulletin, 103, 489–500.

[ece32987-bib-0030] Fu, Y.‐X. (1997). Statistical tests of neutrality of mutations against population growth, hitchhiking and background selection. Genetics, 147, 915–925. 9335623 10.1093/genetics/147.2.915 PMC1208208

[ece32987-bib-0031] Galván‐Tirado, C. , Díaz‐Jaimes, P. , García‐de León, F. J. , Galván‐Magana, F. , & Uribe‐Alcocer, M. (2013). Historical demography and genetic differentiation inferred from the mitochondrial DNA of the silky shark (*Carcharhinus falciformis*) in the Pacific Ocean. Fisheries Research, 147, 36–46.

[ece32987-bib-0032] Goudet, J. (2002). FSTAT: a program to estimate and test gene diversities and fixation indices (version 2.9.3.2). Retrieved from http://www2.unil.ch/popgen/softwares/fstat.htm.

[ece32987-bib-0202] Hare, M. P. , Nunney, L. , Schwartz, M. K. , Ruzzante, D. E. , Burford, M. , Waples, R. S. , … Palstra, F. (2011). Understanding and estimating effective population size for practical application in marine species management. Conservation Biology, 25, 438–449. 21284731 10.1111/j.1523-1739.2010.01637.x

[ece32987-bib-0033] Hastings, A. (1993). Complex interactions between dispersal and dynamics: Lessons from coupled logistic equations. Ecology, 74, 1362–1372.

[ece32987-bib-0034] Heist, E. J. (2008). Molecular markers and genetic population structure of pelagic sharks In Camhi M. D., Pikitch E. K. & Babcock E. A. (Eds.), Sharks of the open ocean: Biology, fisheries and conservation (pp. 323–333). Oxford, UK: Blackwell Publishing, 502 pp.

[ece32987-bib-0203] Heist, E. J. , Musick, J. A. , & Graves, J. E. (1996). Genetic population structure of the shortfin mako (*Isurus oxyrinchus*) inferred from restriction fragment length polymorphism analysis of mitochondrial DNA. Canadian Journal of Fisheries and Aquatic Sciences, 53, 583–588.

[ece32987-bib-0035] Hoelzel, A. R. , Shivji, M. S. , Magnussen, J. , & Francis, M. P. (2006). Low worldwide genetic diversity in the basking shark (*Cetorhinus maximus*). Biology Letters, 2, 639–642. 17148309 10.1098/rsbl.2006.0513 PMC1833978

[ece32987-bib-0036] Hueter, R. E. , & Simpfendorfer, C. A. (2008). Case study: Trends in blue shark abundance in the western North Atlantic as determined by a fishery‐independent survey In Camhi M. D., Pikitch E. K. & Babcock E. A. (Eds.) Sharks of the open ocean: Biology, fisheries and conservation (pp. 236–241). Oxford, UK: Blackwell Publishing, 502 pp.

[ece32987-bib-0037] ICCAT (2005). Report of the 2004 inter‐sessional meeting of the ICCAT sub‐committee on by‐catches: Shark stock assessment. ICCAT Collective Volume of Scientific Papers, 58, 799–890.

[ece32987-bib-0038] ICCAT (2015). Report of the 2015 ICCAT Blue Shark Stock Assessment Session. Retrieved from www.iccat.int.

[ece32987-bib-0039] Jolly, K. A. , da Silva, C. , & Attwood, C. G. (2013). Age, growth and reproductive biology of the blue shark *Prionace glauca* in South African waters. African Journal of Marine Science, 35, 99–109.

[ece32987-bib-0040] Jombart, T. , Devillard, S. , & Balloux, F. (2010). Discriminant analysis of principal components: A new method for the analysis of genetically structured populations. BMC Genetics, 11, 94. 20950446 10.1186/1471-2156-11-94 PMC2973851

[ece32987-bib-0041] Jorde, P. E. , & Ryman, N. (1995). Temporal allele frequency change and estimation of effective size in populations with overlapping generations. Genetics, 139, 1077–1090. 7713410 10.1093/genetics/139.2.1077 PMC1206358

[ece32987-bib-0042] Jorgensen, S. J. , Reeb, C. A. , Chapple, T. K. , Anderson, S. , Perle, C. , Van Sommeran, S. R. , Fritz‐Cope, C. , et al. (2010). Phylopatry and migration of Pacific white sharks. Proceedings of the Royal Society B, 277, 679–688. 19889703 10.1098/rspb.2009.1155 PMC2842735

[ece32987-bib-0043] Keeney, D. B. , & Heist, E. J. (2006). Worldwide phylogeography of the blacktip shark (*Carcharhinus limbatus*) inferred from mitochondrial DNA reveals isolation of western Atlantic populations coupled with recent Pacific dispersal. Molecular Ecology, 15, 3669–3679. 17032265 10.1111/j.1365-294X.2006.03036.x

[ece32987-bib-0044] Keeney, D. B. , Heupel, M. R. , Hueter, R. E. , & Heist, E. J. (2005). Microsatellite and mitochondrial DNA analyses of the genetic structure of blacktip shark (*Carcharhinus limbatus*) nurseries in the nothwestern Atlantic, Gulf of Mexico, and Caribbean Sea. Molecular Ecology, 14, 1911–1923. 15910315 10.1111/j.1365-294X.2005.02549.x

[ece32987-bib-0045] King, J. R. , Wetklo, M. , Supernault, J. , Taguchi, M. , Yokawa, K. , Sosa‐Nishizaki, O. , & Withler, R. E. (2015). Genetic analysis of stock structure of blue shark (*Prionace glauca*) in the north Pacific ocean. Fisheries Research, 172, 181–189.

[ece32987-bib-0046] Kohler, N. E. , Casey, J. G. , & Turner, P. A. (1998). NMFS cooperative shark tagging program, 1962–93: An atlas of shark tag and recapture data. Marine Fisheries Review, 60, 1–87.

[ece32987-bib-0047] Kohler, N. E. , & Turner, P. A. (2008). Stock structure of the blue shark (*Prionace glauca*) in the North Atlantic Ocean based on tagging data In Camhi M. D., Pikitch E. K. & Babcock E. A. (Eds.), Sharks of the open ocean: Biology, fisheries and conservation (pp. 339–350). Oxford, UK: Blackwell Publishing, 502 pp.

[ece32987-bib-0048] Kohler, N. E. , Turner, P. A. , Hoey, J. J. , Natanson, L. J. , & Briggs, R. (2002). Tag and recapture data for three pelagic shark species: Blue shark (*Prionace glauca*), shortfin mako (*Isurus oxyrinchus*) and porbeagle (*Lamna nasus*) in the North Atlantic Ocean. ICCAT Collective Volume of Scientific Papers, 54, 1231–1260.

[ece32987-bib-0049] Leigh, J. W. , & Bryant, D. (2015). popart: Full‐feature software for haplotype network construction. Methods in Ecology and Evolution, 6, 1110–1116.

[ece32987-bib-0050] Lessa, R. , Santana, F. M. , & Hazin, F. H. (2004). Age and growth of the blue shark *Prionace glauca* (Linnaeus, 1758) off northeastern Brazil. Fisheries Research, 66, 19–30.

[ece32987-bib-0051] Li, C. C. , Weeks, D. E. , & Chakravarti, A. (1993). Similarity of DNA fingerprints due to chance and relatedness. Human Heredity, 43, 45–52. 8514326 10.1159/000154113

[ece32987-bib-0052] Librado, P. , & Rozas, J. (2009). DnaSP v5: A software for comprehensive analysis of DNA polymorphism data. Bioinformatics, 25, 1451–1452. 19346325 10.1093/bioinformatics/btp187

[ece32987-bib-0053] Lowe, W. H. , & Allendorf, F. W. (2010). What can genetics tell us about population connectivity? Molecular Ecology, 19, 3038–3051. 20618697 10.1111/j.1365-294X.2010.04688.x

[ece32987-bib-0204] Lynch, M. , & Ritland, K. (1999). Estimation of pairwise relatedness with molecular markers. Genetics, 152, 1753–1766. 10430599 10.1093/genetics/152.4.1753 PMC1460714

[ece32987-bib-0054] Maggs, C. A. , Castilho, R. , Foltz, D. , Henzler, C. , Jolly, M. T. , Kelly, J. , … Viard, F. (2008). Evaluating signatures of glacial refugia for North Atlantic benthic marine taxa. Ecology, 89, sp11. 10.1890/08-0257.1 19097488

[ece32987-bib-0055] Mendonça, F. F. , Ussami, L. H. , Hashimoto, D. T. , Pereira, L. H. , Porto‐Foresti, F. , Oliveira, C. , … Foresti, F. (2012). Identification and characterization of polymorphic microsatellite loci in the blue shark *Prionace glauca*, and cross‐amplification in other shark species. Journal of Fish Biology, 80, 2643–2646. 22650440 10.1111/j.1095-8649.2012.03291.x

[ece32987-bib-0056] Morales, M. J. (2012). Análise genética do tubarão‐raposa Alopias superciliosus no Oceano Atlântico, utilizando região controle do DNA mitocondrial. MSc thesis, Universidade Estadual Paulista – Instituto de Biociências de Botucatu.

[ece32987-bib-0057] Musick, J. A. , Burgess, G. , Cailliet, G. , Camhi, M. , & Fordham, S. (2000). Management of sharks and their relatives (Elasmobranchii). Fisheries, 25, 9–13.

[ece32987-bib-0058] Nakano, H. , & Seki, M. P. (2003). Synopsis of biological data on the blue shark, *Prionace glauca* Linnaeus. Bulletin of the Fisheries Research Agency, 6, 18–53.

[ece32987-bib-0059] Nakano, H. , & Stevens, J. D. (2008). The biology and ecology of the blue shark, *Prionace glauca* In Camhi M. D., Pikitch E. K., & Babcock E. A. (Eds.), Sharks of the open ocean: Biology, fisheries and conservation (pp. 140–151). Oxford, UK: Blackwell Publishing.

[ece32987-bib-0060] Nielsen, E. E. , Wright, P. J. , Hemmer‐Hansen, J. , Poulsen, N. A. , Gibb, I. M. , & Meldrup, D. (2009). Microgeographical population structure of cod *Gadus morhua* in the North Sea and west of Scotland: The role of sampling loci and individuals. Marine Ecology Progress Series, 376, 213–225.

[ece32987-bib-0061] O' Leary, S. J. , Feldheim, K. A. , Fields, A. T. , Natanson, L. J. , Wintner, S. , Hussey, N. , … Chapman, D. D. (2015). Genetic diversity of white sharks, Carcharodon carcharias, in the Northwest Atlantic and southern Africa. Journal of Heredity, 10, esv001. 10.1093/jhered/esv001 25762777

[ece32987-bib-0062] van Oosterhout, C. , Hutchinson, W. F. , Wills, P. M. , & Shipley, P. (2004). MICRO‐CHECKER: Software for identifying and correcting genotyping errors in microsatellite data. Molecular Ecology Notes, 4, 535–538.

[ece32987-bib-0063] Ovenden, J. R. (2013). Crinkles in connectivity: Combining genetics and other types of biological data to estimate movement and interbreeding between populations. Marine and Freshwater Research, 64, 201–207.

[ece32987-bib-0064] Ovenden, J. R. , Kashiwagi, T. , Broderick, D. , Giles, J. , & Salini, J. (2009). The extent of population genetic subdivision differs among four co‐distributed shark species in the Indo‐Australian archipelago. BMC Evolutionary Biology, 9, 1. 19216767 10.1186/1471-2148-9-40 PMC2660307

[ece32987-bib-0065] Palsbøll, P. J. , Berube, M. , & Allendorf, F. W. (2007). Identification of management units using population genetic data. Trends in Ecology and Evolution, 22, 11–16. 16982114 10.1016/j.tree.2006.09.003

[ece32987-bib-0066] Pardini, A. T. , Jones, C. S. , Noble, L. R. , Kreiser, B. , Malcolm, H. , Bruce, B. D. , … Duffy, C. A. (2001). Sex‐biased dispersal of great white sharks. Nature, 412, 139–140. 11449258 10.1038/35084125

[ece32987-bib-0067] Peery, M. Z. , Kirby, R. , Reid, B. N. , Stoelting, R. , Doucet‐Bëer, E. L. , Robinson, S. , … Palsbøll, P. J. (2012). Reliability of genetic bottleneck tests for detecting recent population declines. Molecular Ecology, 21, 3403–3418. 22646281 10.1111/j.1365-294X.2012.05635.x

[ece32987-bib-0068] Petersen, S. L. , Honig, M. B. , Ryan, P. G. , Underhill, L. G. , & Compagno, L. J. (2009). Pelagic shark bycatch in the tuna‐and swordfish‐directed longline fishery off southern Africa. African Journal of Marine Science, 31, 215–225.

[ece32987-bib-0069] Pew, J. , Muir, P. H. , Wang, J. , & Frasier, T. R. (2015). Related: An R package for analysing pairwise relatedness from codominant molecular markers. Molecular Ecology Resources, 15, 557–561. 25186958 10.1111/1755-0998.12323

[ece32987-bib-0070] Pinsky, M. L. , & Palumbi, S. R. (2014). Meta‐analysis reveals lower genetic diversity in overfished populations. Molecular Ecology, 23, 29–39. 24372754 10.1111/mec.12509

[ece32987-bib-0071] Piry, S. , Luikart, G. , & Cornuet, J. M. (1990). BOTTLENECK: A program for detecting recent effective population size reductions from allele data frequencies. Journal of Heredity, 90, 502–503.

[ece32987-bib-0072] Portnoy, D. S. , McDowell, J. R. , Heist, E. J. , Musick, J. A. , & Graves, J. E. (2010). World phylogeography and male‐mediated gene flow in the sandbar shark, *Carcharhinus plumbeus* . Molecular Ecology, 19, 1994–2010. 20406387 10.1111/j.1365-294X.2010.04626.x

[ece32987-bib-0500] Portnoy, D. S. , McDowell, J. R. , McCandless, C. T. , Musick, J. A. , & Graves, J. E. (2009). Effective size closely approximates the census size in the heavily exploited western Atlantic population of the sandbar shark, Carcharhinus plumbeus. Conservation genetics, 10, 1697–1705.

[ece32987-bib-0073] Pratt, H. L. Jr (1979). Reproduction in the blue shark *Prionace glauca* . Fishery Bulletin, 77, 445–470.

[ece32987-bib-0074] Pritchard, J. K. , Stephens, M. , & Donnelly, P. (2000). Inference of population structure using multilocus genotype data. Genetics, 155, 945–959. 10835412 10.1093/genetics/155.2.945 PMC1461096

[ece32987-bib-0075] Queiroz, N. (2010). Diving behaviour, movement patterns and population structure of blue sharks Prionace glauca (L. 1758) in the North–east Atlantic. PhD thesis, University of Aberdeen, Aberdeen, Scotland, U.K. 156 pp.

[ece32987-bib-0076] Queiroz, N. , Humphries, N. E. , Mucientes, G. , Hammerschlag, N. , Lima, F. P. , Scales, K. L. , … Sims, D. W. (2016). Ocean‐wide tracking of pelagic sharks reveals extent of overlap with longline fishing hotspots. Proceedings of the National Academy of Sciences, 113, 1582–1587. 10.1073/pnas.1510090113 PMC4760806 26811467

[ece32987-bib-0077] Queiroz, N. , Humphries, N. E. , Noble, L. R. , Santos, A. M. , & Sims, D. W. (2012). Spatial dynamics and expanded vertical niche of blue sharks in oceanographic fronts reveal habitat targets for conservation. PLoS ONE, 7, e32374. 22393403 10.1371/journal.pone.0032374 PMC3290575

[ece32987-bib-0600] Queller, D. C. , & Goodnight, K. F. (1989). Estimating relatedness using genetic markers. Evolution, 258–275. 28568555 10.1111/j.1558-5646.1989.tb04226.x

[ece32987-bib-0078] R Development Core Team (2016). R: A language and environment for statistical computing. R Foundation for Statistical Computing (Ver. 3.3.2). Retrieved from http://www.R-project.org/.

[ece32987-bib-0079] Rambaut, A. , Suchard, M. A. , Xie, D. , & Drummond, A. J. (2014). Tracer v1.6. Retrieved from http://beast.bio.ed.ac.uk/Tracer.

[ece32987-bib-0080] Randi, E. (2007). Phylogeography of Southern European mammals In Weiss S., & Ferrand N. (Eds.), Phylogeography of Southern European refugia (pp. 101–126). Dordrecht, Netherlands: Springer.

[ece32987-bib-0081] Raymond, M. , & Rousset, F. (1995). Genepop (version 1.2): Population genetics software for exact tests and ecumenicism. Heredity, 86, 248–249.

[ece32987-bib-0082] Rice, J. , Harley, S. , & Kai, M. (2014). Stock assessment of Blue Shark in the North Pacific Ocean using Stock Synthesis. Western and Central Pacific Fisheries Commission Scientific Committee Tenth Regular Session.

[ece32987-bib-0083] Ronquist, F. , Teslenko, M. , van der Mark, P. , Ayres, D. L. , Darling, A. , Höhna, S. , … Huelsenbeck, J. P. (2012). MrBayes 3.2: Efficient Bayesian phylogenetic inference and model choice across a large model space. Systematic Biology, 61, 539–542. 22357727 10.1093/sysbio/sys029 PMC3329765

[ece32987-bib-0084] Rousset, F. (2008). Genepop'007: A complete reimplementation of the Genepop software for Windows and Linux. Molecular Ecology Resources, 8, 103–106. 21585727 10.1111/j.1471-8286.2007.01931.x

[ece32987-bib-0085] Ryman, N. , & Palm, S. (2006). POWSIM: A computer program for assessing statistical power when testing for genetic differentiation. Molecular Ecology, 6, 600–602. 10.1046/j.0962-1083.2001.01345.x 11703649

[ece32987-bib-0086] Schmidt, J. V. , Schmidt, C. L. , Ozer, F. , Ernst, R. E. , Feldheim, K. A. , Ashley, M. V. , & Levine, M. (2007). Low genetic differentiation across three major ocean populations of the whale shark. Rhincodon typus. PloS one, 4, e4988. 10.1371/journal.pone.0004988 PMC2662413 19352489

[ece32987-bib-0087] Schrey, A. W. , & Heist, E. J. (2003). Microsatellite analysis of population structure in the shortfin mako (*Isurus oxyrinchus*). Canadian Journal of Fisheries and Aquatic Sciences, 60, 670–675.

[ece32987-bib-0088] da Silva Ferrette, B. L. , Mendonça, F. F. , Coelho, R. , de Oliveira, P. G. , Hazin, F. H. , Romanov, E. V. , … Foresti, F. (2015). High connectivity of the crocodile shark between the atlantic and southwest Indian oceans: Highlights for conservation. PLoS ONE, 10, e0117549. 25689742 10.1371/journal.pone.0117549 PMC4331560

[ece32987-bib-0089] da Silva, C. , Kerwath, S. E. , Wilke, C. , Meÿer, M. , & Lamberth, S. J. (2010). First documented southern transatlantic migration of a blue shark *Prionace glauca* tagged off South Africa. African Journal of Marine Science, 32, 639–642.

[ece32987-bib-0090] Sippel, T. , Wraith, J. , Kohin, S. , Taylor, V. , Holdsworth, J. , & Taguchi, M. et al. (2011). A summary of blue shark (Prionace glauca) and shortfin mako shark (Isurus oxyrinchus) tagging data available from the North and Southwest Pacific Ocean. ISC/11/SHARKWG‐2/04 Working document submitted to the ISC Shark Working Group Workshop, 28 November–3 December 2011, La Jolla, California USA.

[ece32987-bib-0091] Skomal, G. B. , & Natanson, L. J. (2002). Age and growth of the blue shark, *Prionace glauca*, in the North Atlantic Ocean. ICCAT Collective Volume of Scientific Papers, 54, 1212–1230.

[ece32987-bib-0092] Stevens, J. D. (1990). Further results from a tagging study of pelagic sharks in the north‐east Atlantic. Journal of the Marine Biological Association of the United Kingdom, 70, 707–720.

[ece32987-bib-0093] Stevens, J. D. (2010). Epipelagic oceanic elasmobranchs In Carrier J. C., Musick J. A., & Heithaus M. R. (Eds.), Sharks and their Relatives II: Biodiversity, adaptive physiology and conservation (pp. 3–35). Boca Raton, FL: CRC Press.

[ece32987-bib-0094] Taguchi, M. , King, J. R. , Wetklo, M. , Withler, R. E. , & Yokawa, K. (2015). Population genetic structure and demographic history of Pacific blue sharks (*Prionace glauca*) inferred from mitochondrial DNA analysis. Marine and Freshwater Research, 66, 267–275.

[ece32987-bib-0095] Takahata, N. (1983). Gene identity and genetic differentiation of populations in the finite island model. Genetics, 104, 497–512. 17246145 10.1093/genetics/104.3.497 PMC1202091

[ece32987-bib-0096] Tamura, K. , & Nei, M. (1993). Estimation of the number of nucleotide substitutions in the control region of mitochondrial DNA in humans and chimpanzees. Molecular Biology and Evolution, 10, 512–526. 8336541 10.1093/oxfordjournals.molbev.a040023

[ece32987-bib-0097] Tamura, K. , Peterson, D. , Peterson, N. , Stecher, G. , Nei, M. , & Kumar, S. (2011). MEGA5: Molecular Evolutionary Genetics Analysis using Maximum Likelihood, Evolutionary Distance, and Maximum Parsimony Methods. Molecular Biology and Evolution, 28, 2731–2739. 21546353 10.1093/molbev/msr121 PMC3203626

[ece32987-bib-0098] Tanaka, S. G. , Cailliet, G. M. , & Yudin, K. G. (1990). Differences in growth of the blue shark, *Prionace glauca*: Technique or population. NOAA Technical Report NMFS, 90, 177–187.

[ece32987-bib-0099] Vandeperre, F. , Aires‐da‐Silva, A. , Fontes, J. , Santos, M. , Santos, R. S. , & Afonso, P. (2014). Movements of Blue Sharks (*Prionace* glauca) across their life history. PLoS ONE, 9, e103538. 25119716 10.1371/journal.pone.0103538 PMC4131881

[ece32987-bib-0100] Vandeperre, F. , Aires‐da‐Silva, A. , Lennert‐Cody, C. , Serrão Santos, R. , & Afonso, P. (2016). Essential pelagic habitat of juvenile blue shark (*Prionace glauca*) inferred from telemetry data. Limnology and Oceanography, 61, 1605–1625.

[ece32987-bib-0101] Vandeperre, F. , Aires‐da‐Silva, A. , Santos, M. , Ferreira, R. , Bolten, A. B. , Santos, R. S. , & Afonso, P. (2014). Demography and ecology of blue shark (*Prionace glauca*) in the central North Atlantic. Fisheries Research, 153, 89–102.

[ece32987-bib-0102] Vignaud, T. M. , Maynard, J. A. , Leblois, R. , Meekan, M. G. , Vázquez‐Juárez, R. , Ramírez‐Macías, D. , … Baksay, S. (2014). Genetic structure of populations of whale sharks among ocean basins and evidence for their historic rise and recent decline. Molecular Ecology, 23, 2590–2601. 24750370 10.1111/mec.12754

[ece32987-bib-0700] Wang, J. (2002). An estimator for pairwise relatedness using molecular markers. Genetics, 160, 1203–1215. 11901134 10.1093/genetics/160.3.1203 PMC1462003

[ece32987-bib-0103] Waples, R. S. (1998). Separating the wheat from the chaff: Pattern of genetic differentiation in high gene flow species. Journal of Heredity, 89, 438–450.

[ece32987-bib-0104] Waples, R. S. , Luikart, G. , Faulkner, J. R. , & Tallmon, D. A. (2013). Simple life‐history traits explain key effective population size ratios across diverse taxa. Proceedings of the Royal Society of London B: Biological Sciences, 280, 20131339. 10.1098/rspb.2013.1339 PMC3757969 23926150

[ece32987-bib-0105] White, T. A. , Fotherby, H. A. , Stephens, P. A. , & Hoelzel, A. R. (2010). Genetic panmixia and demographic dependence across the North Atlantic in the deep‐sea fish, blue hake (*Antimora rostrata*). Heredity, 106, 690–699. 20717157 10.1038/hdy.2010.108 PMC3183912

