# Supplementary material for: World without borders—genetic population structure of a highly migratory marine predator, the blue shark (Prionace glauca)
Source: Ecol Evol. 2017 May 24;7(13):4768–81. doi: 10.1002/ece3.2987 (PMC5496551; doi:10.1002/ece3.2987)
Supplement: Supplementary file 1 [file ECE3-7-4768-s001.pdf]

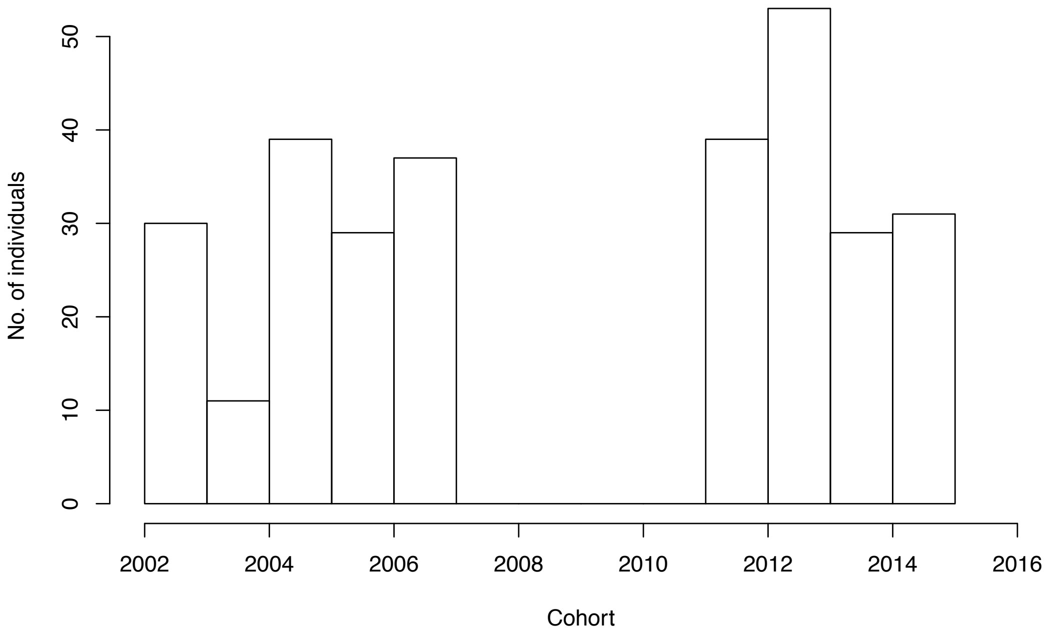

Supplementary Figure 1 – Cohort composition of the two temporal groups of blue sharks from all Atlantic nursery areas. Group 2000s – individuals sampled from cohorts 2003-2008; group 2010s - individuals sampled from cohorts 2012-2015.

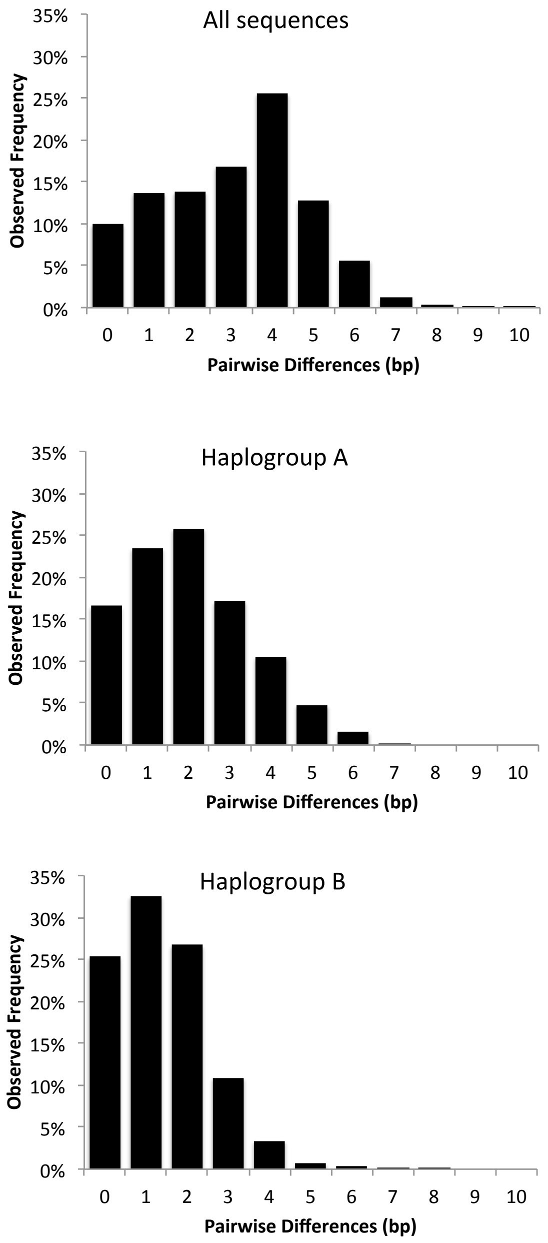

Supplementary Figure 2 – Distribution of pairwise differences among blue shark mtDNA control region haplotypes, considering all sequences together, only haplogroup A, and only haplogroup B sequences.

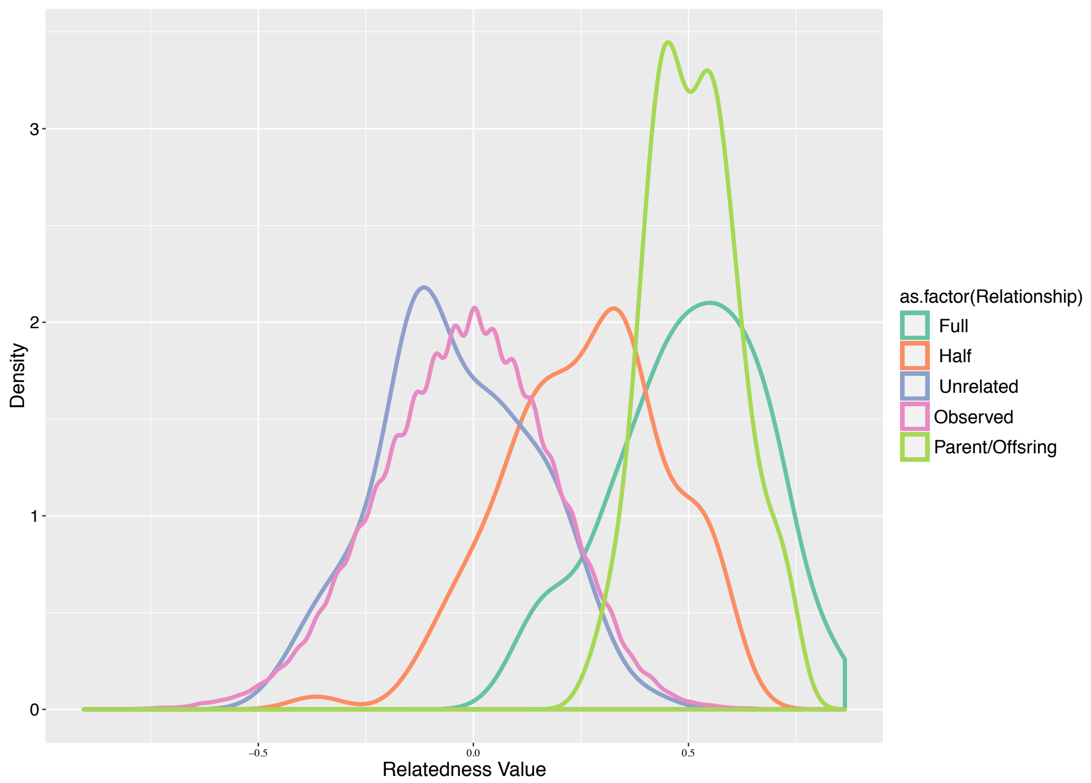

Supplementary Figure 3 – Distribution of pairwise genetic relatedness values for simulated pairs of individuals, i.e. Unrelated, Parent/Offspring, Full siblings, Half siblings), and for all observed pairs of individuals sampled within Atlantic nursery areas.

A) Eleven loci and Atlantic nursery sample collections

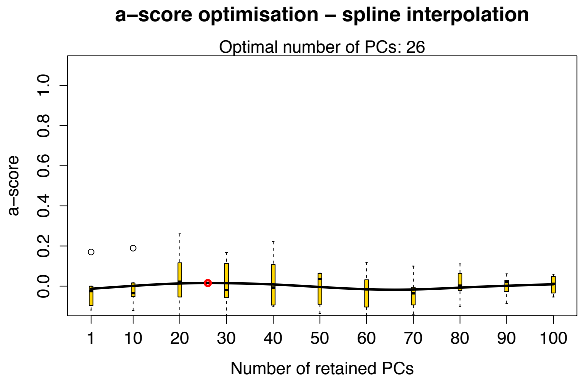

B) Six loci and all Atlantic sample collections

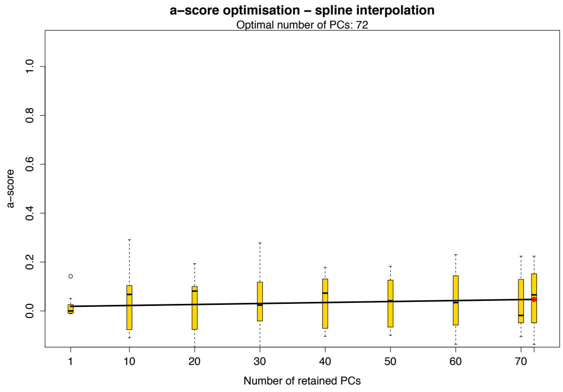

Supplementary Figure 4 – Plot of alpha-scores from the Discriminant Analysis of Principal Components using A) eleven microsatellite loci and only the three Atlantic nursery sample collections, and B) using six microsatellite loci and all Atlantic samples collections.

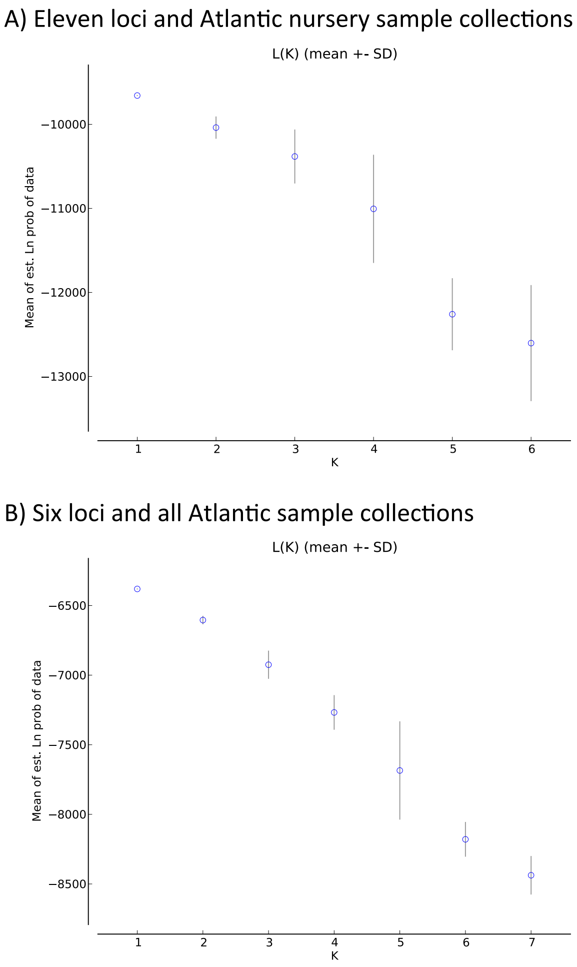

Supplementary Figure 5 – Likelihood of K values from the STRUCTURE runs, based on A) eleven loci and only Atlantic nursery sample collections, and B) six loci and all Atlantic samples collections.

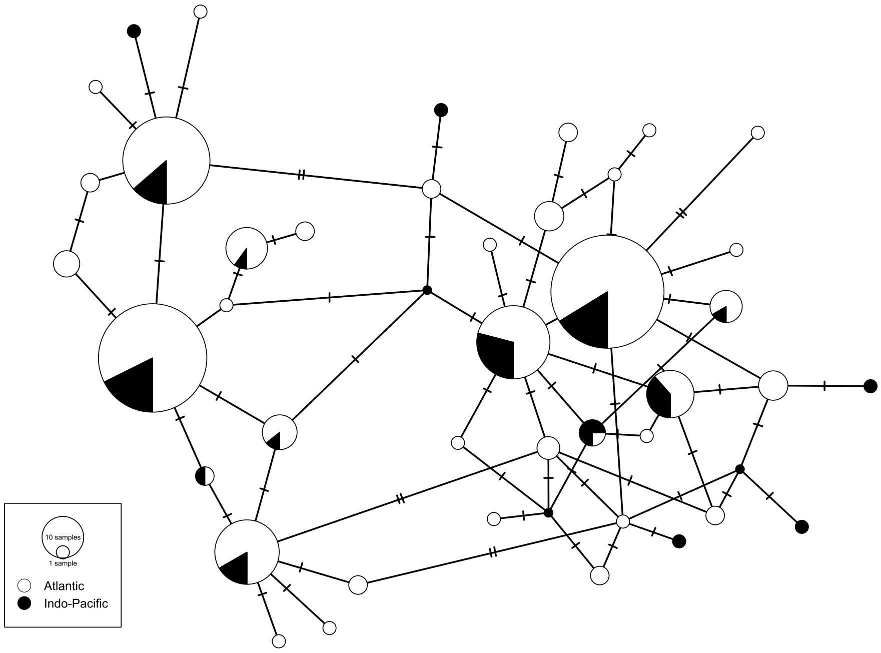

Supplementary Figure 6 - Mitochondrial control region haplotype network based on Atlantic and Indo-Pacific blue shark sample collections. Number of mutated positions among connected haplotypes in the network are indicated by the slash marks.
